# Distribution models calibrated with independent field data predict two million ancient and veteran trees in England

**DOI:** 10.1002/eap.2695

**Published:** 2022-08-09

**Authors:** Victoria Nolan, Francis Gilbert, Tom Reed, Tom Reader

**Affiliations:** ^1^ Life Sciences University of Nottingham Nottingham UK; ^2^ Woodland Trust Grantham UK

**Keywords:** ancient trees, bias correction, conservation, sampling bias, species distribution modeling, veteran trees, zero‐inflated

## Abstract

Large, citizen‐science species databases are powerful resources for predictive species distribution modeling (SDM), yet they are often subject to sampling bias. Many methods have been proposed to correct for this, but there exists little consensus as to which is most effective, not least because the true value of model predictions is hard to evaluate without extensive independent field sampling. We present here a nationwide, independent field validation of distribution models of ancient and veteran trees, a group of organisms of high conservation importance, built using a large and internationally unique citizen‐science database: the Ancient Tree Inventory (ATI). This validation exercise presents an opportunity to test the performance of different methods of correcting for sampling bias, in the search for the best possible prediction of ancient and veteran tree distributions in England. We fitted a variety of distribution models of ancient and veteran tree records in England in relation to environmental predictors and applied different bias correction methods, including spatial filtering, background manipulation, the use of bias files, and, finally, zero‐inflated (ZI) regression models, a new method with great potential to investigate and remove sampling bias in species data. We then collected new independent field data through systematic surveys of 52 randomly selected 1‐km^2^ grid squares across England to obtain abundance estimates of ancient and veteran trees. Calibration of the distribution models against the field data suggests that there are around eight to 10 times as many ancient and veteran trees present in England than the records currently suggest, with estimates ranging from 1.7 to 2.1 million trees compared to the 200,000 currently recorded in the ATI. The most successful bias correction method was systematic sampling of occurrence records, although the ZI models also performed well, significantly predicting field observations and highlighting both likely causes of undersampling and areas of the country in which many unrecorded trees are likely to be found. Our findings provide the first robust nationwide estimate of ancient and veteran tree abundance and demonstrate the enormous potential for distribution modeling based on citizen‐science data combined with independent field validation to inform conservation planning.

## INTRODUCTION

Citizen‐science species databases and other large species record collections are becoming increasingly useful in conservation research and planning and are able to provide a great deal of information about species distributions across large geographical areas and time periods (Pearce & Boyce, 2006; Schmeller et al., 2009; Tiago, Pereria, et al., 2017). Nevertheless, sampling bias (also known as sample selection or survey bias) in this sort of species data is a widely acknowledged problem (Hijmans, 2012; Phillips et al., 2009; Syfert et al., 2013). Sampling bias results in certain areas or species being sampled more intensively or frequently, most commonly because of issues relating to accessibility and the location of the recorders, for example, travel time from a recorder's home to a survey site (Dennis & Thomas, 2000), distance from roads or the availability of pathways (Kadmon et al., 2004; Reddy & Dávalos, 2003; Schulman et al., 2007), or elevation/terrain steepness (Mair & Ruete, 2016). The selective surveying of rare, “special” species or interesting geographic areas also generates sampling bias in species data (Kramer‐Schadt et al., 2013; Reddy & Dávalos, 2003; Snäll et al., 2011). Quantifying bias is further complicated by the fact that different taxa suffer from different causes of spatial bias (Mair & Ruete, 2016).

Species distribution modeling (SDM) is a common and effective tool for understanding and predicting species distributions and distributional shifts (Beaumont et al., 2007; Chen et al., 2011; Clement et al., 2014). SDM works by assessing the known presence (and sometimes absence) records of a species in relation to environmental variables. The suitability of locations for this species, reflecting its fundamental niche and geographic range, can then be predicted based on environmental characteristics (Araújo & Guisan, 2006; Hijmans & Graham, 2006; Mateo et al., 2011). Many modeling techniques are available, with maximum entropy (MaxEnt) modeling being by far the most widely used because of its ability to use presence‐only data and to cope with small data sets (Elith et al., 2006; Hernandez et al., 2006; Phillips et al., 2006). Sampling bias in species data can greatly influence SDM performance and quality because it leads to an exaggeration of the importance of environmental conditions for species in better surveyed locations (Syfert et al., 2013; Stolar & Nielsen, 2015). Therefore, predicted species distributions from models built with biased records can vary dramatically compared to the actual distribution; the predictions partly represent survey effort rather than species niche requirements (Phillips et al., 2009). Incorrect model predictions are particularly detrimental in the planning of conservation projects and decision‐making about which areas should be protected or subjected to management (MacKenzie, 2005). Various methods to assess and correct for sampling bias have been developed recently, and issues created by sampling bias in SDM and citizen‐science recording schemes are now widely recognized (Boria et al., 2014; Fourcade et al., 2014; Kramer‐Schadt et al., 2013; Phillips et al., 2009). However, thorough evaluations of these methods using independently collected, unbiased species data are scarce, and the true value of many distribution models built using biased data remains unclear.

Ground‐truthing of model verifications using independently collected, unbiased new data is the ideal scenario when testing model performance and predictions, yet distribution models are rarely tested in this way (Costa et al., 2010; Fabri‐Ruiz et al., 2019; Greaves et al., 2006). The reasons for this are obvious since the time and financial cost of large‐scale surveys is often prohibitive and difficult. However, the networks of volunteer recorders for many citizen‐science projects may lend themselves to planned ground‐truthing, and with some forward planning, robust, strategic sampling methods could be applied in many of these large projects. In this study we use a large volunteer survey network of a nationwide citizen‐science project, the UK Ancient Tree Inventory (ATI), to do just that: By recruiting a sample of enthusiastic volunteers who regularly record for the project, we carried out nationwide, randomized surveys in order to validate model predictions independently using the newly collected unbiased species data, with the aim of selecting the most robust predictive models of species distributions.

Dead and decaying wood ecosystems are highly complex and fragile and are found worldwide (Butler et al., 2002; Hodge & Peterken, 1998; Seibold & Thorn, 2018; Siitonen, 2001). They provide resources and habitats for numerous threatened and endangered saproxylic species (Jonsson et al., 2005; Seibold et al., 2015). Ancient and veteran trees (sometimes also known as large, old trees or heritage trees) are trees that are in the later stages of their life phase and exhibit “veteran characteristics” such as a retrenched crown, hollowing trunk, holes, and cavities (ATF, 2008; Nolan et al., 2020; Read, 2000). Strict definitions of “ancient” and “veteran” do not exist globally, and the terms are often used interchangeably (Nolan et al., 2020). However, in the UK all trees that show veteran characteristics are usually classed as veteran, and within this classification, only trees that are significantly older than most individuals of the same species (often based on girth measurements) are classed as ancient (ATF, 2008; Nolan et al., 2020).

Ancient and veteran trees are essential contributors to the persistence of dead and decaying wood ecosystems in most biomes (Humphrey, 2005; Read, 2000; Speight, 1989) and provide insights into past landscape use and management and important historical events (Nolan et al., 2020; Rackham, 1976; Read, 2000; Zhang et al., 2017). Nevertheless, ancient and veteran trees are declining around the world (Fischer et al., 2010; Gibbons et al., 2008; Kirby & Watkins, 2015; Roux et al., 2014), which is attributed to urbanization, agricultural intensification, and a lack of planting, management, and awareness of the development of ancient and veteran tree populations (ATF, 2005, 2011; Fay, 2002; Lindenmayer et al., 2012; Lonsdale, 2013; Read, 2000). In addition, relatively few countries have knowledge about or monitor ancient and veteran trees sufficiently well for conservation measures to be effective (Nolan et al., 2020).

The UK is unique in having excellent records of ancient and veteran trees. The ATI (formerly known as the Ancient Tree Hunt) is a national database of over 200,000 ancient, veteran, and other noteworthy trees (Nolan et al., 2020). The ATI is a great example of a successful and popular citizen‐science project, with hundreds of new tree records uploaded to the online inventory managed by the Woodland Trust each month by members of the public, ecological organizations, and specialized ancient tree volunteer recorders. Nevertheless, like many citizen‐science projects and online species databases, because of the nonrandom, unstructured nature of the recording process, there is likely to be a high level of sampling bias in the ATI. Therefore, the current distribution map of ancient and veteran trees based on the ATI may be more reflective of recorder activity in certain locations than it is of the true geographical distribution of trees. It is also likely that there is huge under‐recording of trees in many areas, especially those that are less accessible, less interesting to survey, or farther away from centers of human population (Mair & Ruete, 2016; Phillips et al., 2009). Thus, despite the large number of records collected, there are thought to be many more undiscovered ancient and veteran trees in the UK, including those that are at risk of damage or destruction (Nolan et al., 2020). Obtaining insight into the true distribution of ancient and veteran trees, as well as under‐ or well‐surveyed areas (i.e., patterns of sampling bias), is therefore key for the conservation and protection of this important component of biodiversity.

Another problem with using nonrandomly sampled species data, as found in the ATI, that is often encountered in SDM is the lack of information about true absences—locations where the species is definitively not present, as opposed to those that have simply not been surveyed (Hastie & Fithian, 2013). Presence‐only SDM most commonly overcomes this by generating “pseudo‐absence” points across the study area. These points are usually positioned at random (Stockwell & Peters, 1999), but they can be weighted by geography, environment, or target group sampling (Hirzel et al., 2001; Phillips & Dudík, 2008). However, the method of pseudo‐absence generation has been shown to influence model outcomes (Barbet‐Massin et al., 2012; Wisz & Guisan, 2009) and can result in unreliable models (Liang et al., 2018).

Predictive species distribution maps based on abundance are much less common than those based on presence or presence–absence, because most large species data sets record only species occurrence (Lyashevska et al., 2016). If the spatial predictors in SDM are only available at a greater resolution than the occurrence data, occurrences must be aggregated to presence‐only or presence–absence at the same resolution, which results in a loss of vital information about species density across the study area (Johnston et al., 2015; Nolan, Gilbert, & Reader, 2021). An alternative to aggregating occurrences to presence–absence data is to aggregate them into counts of occurrences (i.e., abundance or pseudo‐abundance) at the resolution of the spatial predictors, an approach that retains information about species density and can produce better fitting, more accurate predictive maps (Howard et al., 2014; Johnston et al., 2015; Nolan, Gilbert, & Reader, 2021). One problem with this method is that the new aggregated abundance data are highly likely to be zero‐inflated (ZI) compared with the standard distributions that they are typically expected to follow (Bird et al., 2014; Martin et al., 2005), but this can be overcome with the use of ZI models (Lambert, 1992). ZI models, which have received relatively little attention in the field of SDM, are able to cope with such data and have been shown to be able to both identify causes of sampling bias and facilitate its removal in simulated species data (Nolan, Gilbert, & Reader, 2021). Here we use our ATI case study to test our recently proposed method of sampling bias correction using ZI models (Nolan, Gilbert, & Reader, 2021).

The aim of this study was to produce the best possible unbiased prediction of the current distribution of ancient and veteran trees in England using distribution modeling and large‐scale field validation. This work builds on previously published research that used only subsets of the ATI to make inferences about ancient and veteran tree abundance and distributions across smaller geographical areas of the UK or specific types of habitat (Nolan et al., 2020; Nolan, Reader, et al., 2021). Our work presented here is the first to use all ancient and veteran tree records in England across a continuous landscape. In previous work (Nolan, Reader, et al., 2021), we attempted to validate models using historical maps, but here we go a step further and collect new field data. This enables us to validate the model fully and presents an interesting opportunity to evaluate independently the effectiveness of a variety of bias correction methods in relation to our distribution models, which is something that relatively few studies attempt. We fit distribution models with a variety of different bias correction methods, including ZI models, and evaluate their performance and predictive power using both common internal model validation methods and our independently collected, unbiased field estimates of ancient and veteran tree abundance. Thorough, independent evaluation of the most robust, accurate predictive maps of ancient and veteran tree distribution can assist with future targeted surveys, provide estimates of the work needed to find undiscovered trees to add to the ATI for their protection, and help to estimate the landscape‐scale biological value of this habitat‐rich resource as a whole.

## METHODS

### Study species and environmental predictors

**FIGURE 1:**
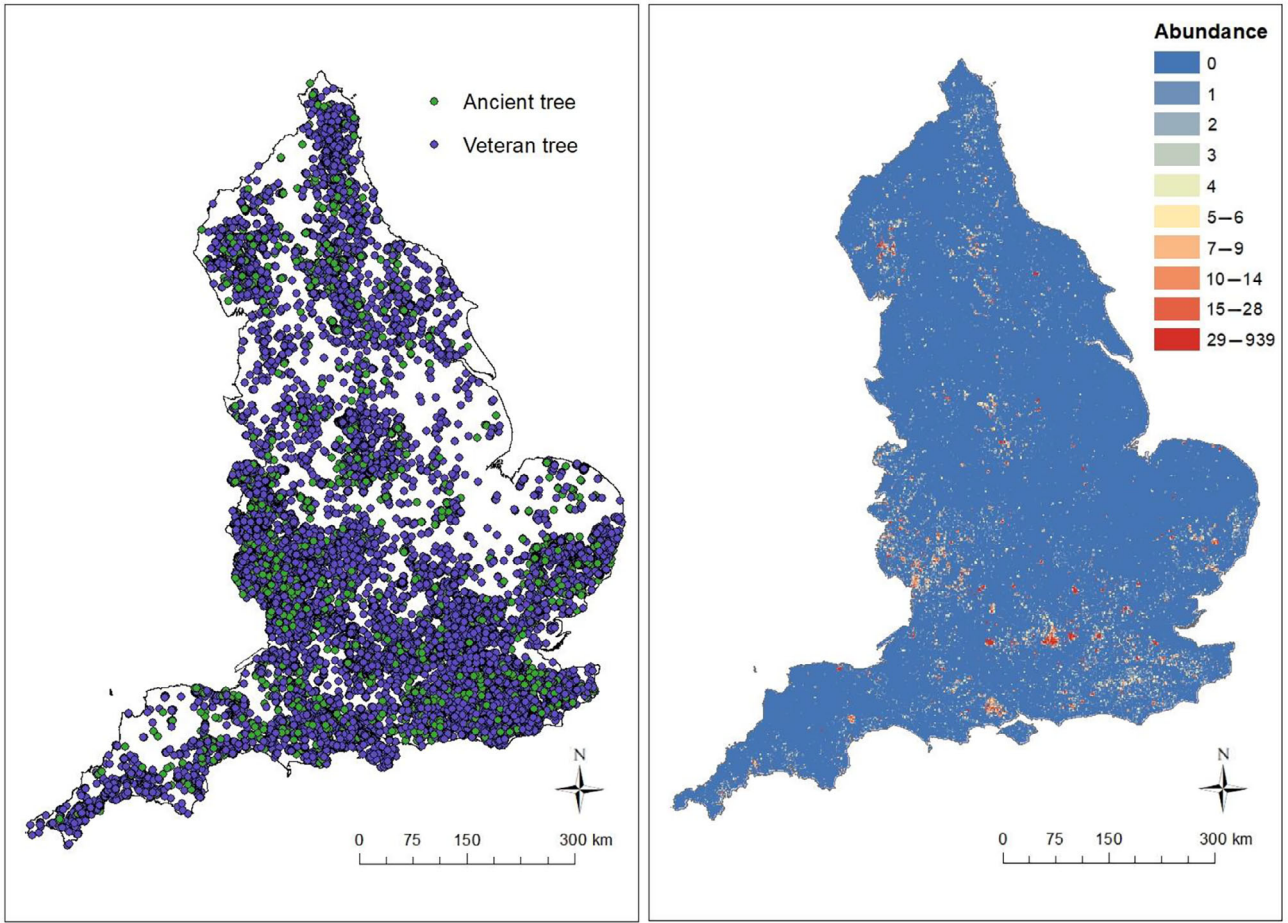
Left: Ancient and veteran tree records across England from Ancient Tree Inventory (ATI). There are 94,024 records in total (10,450 ancient, 83,574 veteran). Right: Ancient and veteran tree record abundance (counts of records) per 1‐km grid square. Abundance ranges from 0 (blue) to 939 (red).

A grid consisting of 130,754 cells with a resolution of 1 × 1 km was created within the boundaries of England: This was the maximum total number of grid cells possible that fell completely within the boundaries. Records were obtained from the ATI (accessed 17 December 2018) and were queried and selected as being of “ancient” or “veteran” status located in the country “England.” All years were included up to the access date (December 2018). In addition, the ATI has a thorough record verification process consisting of two steps. First, once uploaded by a recorder, each record is required to be revisited by a trained verifier to check the record location and associated information. Based on this, each record comes with an additional rating (Appendix S1: Table S1) concerning its reliability by the ATI managers at the Woodland Trust (Nolan et al., 2020). We queried and excluded all records with a rating below three stars, meaning they are unverified and potentially unreliable. This left 93,404 ancient and veteran tree records within our generated grid in England (Figure 1), which composed our final species data set used throughout the rest of the analysis. Twenty environmental, topographical, and anthropogenic characteristics were then collected across the study area for each 1‐km grid cell (Table 1). Four predictors were categorical (agricultural class, land class, soil type, and type of historic countryside) and 16 were numeric. See Appendix S1: Tables S2–S5 for a full explanation of each categorical variable. No strong correlations were found between any pair of numeric predictors (Pearson's correlation coefficient threshold ±0.6, variance inflation factor [VIF] < 5). Each predictor was converted to raster format at a 1‐km resolution. All processing of predictors was carried out in ArcGIS version 10.3.1 (ESRI, 2018). For a full visual summary of the complete methodology and the subsequently described bias correction and modeling processes see Appendix S1: Figure S1.

**TABLE 1:** All 20 predictor variables for each grid cell, along with the type of data, source, and date the data were accessed. There are 16 continuous predictors and four categorical predictors.

Predictor	Type	Source (date accessed)	Justification for inclusion
Historical predictor			
Distance from historic forest (km)	Numeric	Neilson, 1940 in The English Government at Work (Willard and Morris, 1940)—The Forests: 1327–1336 (2 July 2018)	In the UK certain types of historic sites such as these are thought to be less likely to have been deforested, and their ancient trees are more likely to have been protected than in the wider countryside (Farjon, 2017; Rackham, 1976), particularly due to their continuous royal or aristocratic ownership over the centuries (Butler et al., 2002).
Distance from moated site (km)	Numeric	Aberg, 1978—Medieval moated sites (5 July 2018)	
Distance from medieval deer park (km)	Numeric	Rackham, 1976—Trees and Woodland in the British Landscape (5 July 2018)	
Distance from Tudor deer park (km)	Numeric	The Counties of Britain: A Tudor Atlas by John Speed (Nicolson and Hawkyard, 1989)—(3 July 2018)	
Type of countryside	Categorical	Rackham, 1976—Trees and Woodland in the British Landscape (5 July 2018)	The divisions in the historical landscape are likely to influence the management and persistence of tree populations (Rackham, 1976). See Appendix S1: Table S5.
Topographical predictor			
Distance from a water course (km)	Numeric	OS Open Rivers V.10/2018 (Vector) (7 January 2019)	Environmental characteristics such as these shape the microclimate experienced by the trees throughout their whole lives and are likely to influence the species composition, dispersal, decay, and other dynamics of ancient and veteran tree populations (Hall & Bunce, 2011; Hartel et al., 2018; Williamson et al., 2017).
Mean altitude (m)	Numeric	Altitude (elevation above sea level in meters)—WorldClim DEM (10 May 2018)	
Most common soil type	Categorical	EU Soil Database—World Reference Base (WRB) for Soil Resources full soil code (WRBFU) (24 September 2018)	
Anthropogenic predictor			
Distance from nearest town center (km)	Numeric	Government Open Data—English Town Centres 2004 (19 March 2018)	The presence of ancient and veteran trees across the UK landscape has experienced strong human influences across many centuries (Farjon, 2017; Rackham, 1976; Williamson et al., 2017). Therefore, it is likely that proximity to towns, cities, and roads would have shaped the planting and management of ancient and veteran trees. Additionally, many of these characteristics also are likely to influence ancient and veteran tree sampling due to issues around accessibility, favoring certain sites, and so forth (Mair & Ruete, 2016; Reddy & Dávalos, 2003).
Distance from nearest major city (km)	Numeric	Office of National Statistics (ONS)—Major Towns and Cities 2015 (29 November 2017)	
Distance from commons (km)	Numeric	Government Open Data—Commons register 2015 (18 December 2018)	
Distance from major road (km)	Numeric	Government Open Data—Major Road Network 2016 (5 November 2017)	
Length of minor roads (km)	Numeric	OS Open Map Local V.10/2018 (Vector)—Road (7 January 2019)	
Land classification predictor			
Cover of ancient woodland (%)	Numeric	Natural England—Ancient Woodlands (England) inventory (8 January 2018)	Ancient and veteran trees can sometimes be found in woodland (especially ancient woodland [Peterken, 1977]) and so could be an important habitat in which they are present (Lonsdale, 2013).
Cover of forest or woodland (%)	Numeric	Government Open Data—National Forest Inventory (NFI) 2016 (4 December 2017)	
Cover of traditional orchard (%)	Numeric	Natural England—Traditional Orchards HAP England (10 January 2018)	Ancient and veteran trees have strong connections to wood‐pasture habitat (Hartel et al., 2013) and traditional orchards (Williamson et al., 2017).
Cover of wood pasture (%)	Numeric	Natural England—Wood Pasture and Parkland BAP Priority Habitat Inventory (4 December 2017)	
Distance from nearest National Trust site (km)	Numeric	National Trust—Open data: limited access land and always open land (8 January 2019)	The National Trust is a large organization in the UK that holds vast areas of land with historic or natural interest and therefore have strong links to ancient and veteran trees (Nolan et al., 2020).
Most common agricultural classification	Categorical	Natural England—Provisional Agricultural Land Classification England 2013 (13 April 2018)	Land‐use change, agricultural intensification, and urbanization represent significant influences on ancient and veteran tree decline around the world (ATF, 2005, 2011; Fay, 2002; Lonsdale, 2013; Read, 2000). In addition, tree populations have specialized niche requirements to grow and survive and are therefore likely to be adapted to particular environmental conditions relating to specific land types (Williamson et al., 2017).
Most common land classification	Categorical	Centre for Ecology and Hydrology (CEH)—Land Cover Map 2015 (LCM2015, 1 km dominant target class) (29 March 2017)	

### Bias correction techniques

Four types of bias correction method were tested, three of which are conventional presence‐only or presence–absence SDM techniques previously used and evaluated (Beck et al., 2014; Fourcade et al., 2014; Kramer‐Schadt et al., 2013). These were (1) spatial filtering of occurrence records, (2) restriction of the selection of pseudo‐absence background data, and (3) the use of bias files in the models (Table 2). Three methods of spatial filtering were tested, the first of which was systematic sampling (Beck et al., 2014; Fourcade et al., 2014), where grids with resolutions of 2, 5, and 10 km were created with the same extent as that of the occurrence records. One occurrence record was then randomly sampled from each 2‐, 5‐, and 10‐km grid cell, resulting in a filtering of occurrence records from a total of 94,024 to 11,261, 5504, and 2495 final occurrence records, respectively.

**TABLE 2:** Types of bias correction method applied to ancient tree inventory records when modeling the distribution of ancient and veteran trees across England.

Method	Type	Description
Spatial filtering	Systematic sampling	Randomly sampling one occurrence point per grid cell of 2‐, 5‐, or 10‐km resolution
	Cluster analysis	Randomly sampling one occurrence point per grouped cluster of records within distance of 1 km
	Weighted distance	Sample 20,000 occurrence points based on weighted probabilities of distance to nearest other occurrence location. Occurrences with greater distances to other occurrence locations were more favored in the selection process
Background restriction	Restricting background selection area	Restricting the area within which pseudo‐absences are randomly chosen by creating buffers at varying distances (1, 2, 5, and 10 km) around each occurrence location. Pseudo‐absences generated were then confined solely to these areas
Bias files	Recorder location	Weighted probability surface for selection of 10,000 pseudo‐absence points based on kernel density analysis of locations of recorder home bases (centroid locations of all records uploaded by each recorder)
	Density of towns and cities	Weighted probability surface for selection of 10,000 pseudo‐absence points based on kernel density analysis of locations of all town and city centroids across England
	Density of roads (major and minor)	Weighted probability surface for selection of 10,000 pseudo‐absence points based on kernel density analysis of all major and minor roads across England
	Altitude	Weighted probability surface created by rescaling altitude values at 1‐km resolution across England for selection of 10,000 pseudo‐absence points
	Distance to nearest of wood pasture	Weighted probability surface for selection of 10,000 pseudo‐absence points based on 1‐km resolution raster layer of distance to nearest wood‐pasture across England
	Record density (abundance of records per 1‐km grid cell)	Weighted probability surface for selection of 10,000 pseudo‐absence points based on record density per 1‐km grid cell (i.e., abundance of ancient and veteran tree records)
Zero‐inflated (ZI) regression models	Use of “pseudo‐abundance”	Aggregating presence records to a count of “pseudo‐abundance” at a resolution of 1 km and fitting ZI models to identify and correct for sampling bias (Nolan, Gilbert, & Reader, 2021); predictions of abundance for each grid cell can be used to create distribution map of ancient and veteran trees across England

The second method was “cluster analysis” (Fourcade et al., 2014), whereby all occurrence records within 1 km of each other were grouped together as a single cluster. Thus, some records in the same cluster were more than 1 km from each other, but all were <1 km from at least one other record in the cluster. From each cluster a single occurrence record was randomly selected and retained. All records that were farther than 1 km from the next record and did not fall within a cluster were also retained, resulting in a final total of 1583 occurrence records. The final spatial filtering method was “weighted distances” (Boria et al., 2014; Kramer‐Schadt et al., 2013; Veloz, 2009), where the distance of the nearest record was calculated for each occurrence location and rescaled into a probability of weighted distances between 0 and 1. A total of 20,000 occurrence records were then selected based on these weighted probability distances, so that records with large distances to the nearest other record were more likely to be selected (i.e., had a probability closer to 1). The processing of the occurrence records for each of these three filtering methods was carried out manually in R (R Core Team, 2018) and ArcGIS.

The other two bias correction methods are both types of manipulation of the selection of the pseudo‐absences from the background when fitting distribution models but differ based on their requirements. The first method, background restriction (Table 1), requires no prior knowledge of sampling bias but involves restricting the area within which the pseudo‐absence data were selected (Fourcade et al., 2014; Phillips, 2008). This was done by creating buffer areas around each occurrence point at distances of 1, 2, 5, and 10 km, within which the pseudo‐absence selection was confined. The second method employs bias files, which are proxies of likely sources of bias across the study area (Dudík et al., 2005; Elith et al., 2010). The bias file is used to influence the weighted selection of pseudo‐absence locations. Six different potential bias sources were considered (Table 1). Two of these bias files were record density (number of trees per grid square) and recorder density (centroid location of each recorder's specific records). Having access to information about recorder locations allows us to examine true factors that cause sampling bias rather than just environmental proxies, which is something that many large databases are unable to do.

The fourth bias correction method is a novel approach we recently developed (Nolan, Gilbert, & Reader, 2021), whereby the 93,404 presence‐only ATI records were aggregated into a count of occurrences per 1‐km grid cell (“abundance”) (Figure 1). In some cases it is likely that this abundance measure is more likely pseudo‐abundance, as in many species databases single occurrences represent the presence of multiple individuals at a single location. With the ATI data this is less likely to be the case because each tree is recorded as a single individual, so we use the term abundance throughout, although we acknowledge that pseudo‐abundance may be more appropriate in other cases. This results in 12,687 cells (9.7%) containing one or more records, with abundance ranging from 0 to 939 trees per 1‐km grid cell. Aggregating to count data allowed ZI models to be fitted to the pseudo‐abundance data and used to both identify and correct for sampling bias (Nolan, Gilbert, & Reader, 2021.).

### Modeling and analysis

MaxEnt presence‐only models were fitted to the 93,404 ancient and veteran tree occurrence records under each of the presence‐only bias correction methods (spatial filtering, background manipulation and bias files) at a 1‐km resolution using the ENMeval package in R (Muscarella et al., 2014). An additional model with no bias correction (i.e., the raw occurrence data) was also fitted for comparison. All models were fitted using 10,000 pseudo‐absence background points, which were randomly sampled across the study area unless explicitly different due to the bias correction method. All other MaxEnt parameters were left at their default values (Phillips & Dudík, 2008). Model tuning was also undertaken, and the best fitting model for each bias correction method selected based on the corrected Akaike information criterion (AIC_c_) (Appendix S2: Section S2). All 20 predictors (Table 1) were used for each model, but for models using bias files based on one or more of the predictors (towns and cities, roads, altitude, or wood‐pasture bias files), models were fitted both with and without those particular predictors for comparison.

Model predictions were created for each MaxEnt model and evaluated using 10‐fold cross‐validation (CV), where the data are randomly split into 10 parts, with each part sequentially acting as the “test” data during internal model evaluation, while the other nine are used to train the model. Initial analysis (not shown) was used to evaluate alternative nonrandom methods of geographically splitting the data into training and test data, but these proved less effective than CV (Appendix S2: Figure S1). Models were evaluated using AIC_c_ and area under the curve (AUC) for the training and test data. AIC_c_ is a test of model fitting and performance based on goodness of fit and its ability to avoid overfitting and can be used to compare between the fit of different models (Akaike, 1973). AUC, on the other hand, is a measure of a model's predictive power based on the ROC (receiver operating characteristic) curve and its ability to correctly classify observations across all possible thresholds of classification of the probability of presence (Fielding & Bell, 1997; Lobo et al., 2008). AUC has been criticized as an evaluation metric of distribution modeling (Lobo et al., 2008; Peterson et al., 2008), yet it remains one of the most widely used evaluation methods in SDM.

For the fourth bias correction method (ZI models), ZI models were fitted to the pseudo‐abundance data. ZI models are an extension of generalised linear models (GLMs) and combine two components: (1) a zero component, which models the probability that an observation is an excess zero, and (2) a count component, which produces the count estimates (Lambert, 1992; Welsh et al., 1996; Zuur et al., 2009). Because there are two parts, processes generating true zeroes and excess (potentially false) zeroes can be modeled separately (Zuur et al., 2009). When used for SDM with species abundance data suffering from sampling bias, the zero component can model the probability that an abundance of zero at a particular location is truly zero or not, and the count component can then produce an estimate of true abundance at that location (Nolan, Gilbert, & Reader, 2021). Therefore, ZI models have great potential to model geographically biased species data and to allow examination of the sources of bias, if unknown, as well as producing predictive SDM maps free of bias. Several studies have used ZI models to examine sampling bias in species data (Dwyer et al., 2016; Tiago, Ceia‐Hasse, et al., 2017; Williams et al., 2016), but none has used this method to produce prediction maps from real species data.

ZI models were fitted with either a Poisson or negative binomial (NB) distribution. Both error distributions are commonly used for count data and can be applied within a ZI model framework (Zuur et al., 2009). A NB distribution allows for more overdispersion in the data than the Poisson distribution and can account for some (but often not all) of the excess zeroes in ZI data sets through the use of an extra parameter (ϴ) (Fisher, 1941). Therefore, it may be more appropriate to use this distribution if there is biological aggregation in the data (Lindén & Mäntyniemi, 2011). However, it is important to note there can be other causes of overdispersion in ecological data, such as the scaling of nonhomogeneous processes or nonindependence in the data (Richards, 2007). In our case, the pseudo‐abundance data show huge overdispersion, indicated by a variance: mean ratio of 122.7 (with ratios over 1 suggesting overdispersion). Therefore, it is likely that a NB distribution will be more appropriate, even if there is still zero inflation. The performance of each model was compared using Vuong's AIC_c_ test for nonnested models (Vuong, 1989).

All environmental predictors were included as main, linear effects in both components (count and zero) of the ZI models in order to examine both the potential influence of each predictor on both species' ecology and sampling behavior (Table 1). All numeric predictors were centered and scaled. Several categories from the categorical variables soil type, agricultural class, and land class were combined to aid model fitting. Therefore, there were three agricultural classes (agricultural, nonagricultural, and other), 10 land classes (arable, grassland, urban, coniferous, coastal, freshwater, saltwater, heather/bog, broadleaved, and other), and 10 soil types (luvisol, cambisol, gleysol, fluvisol, podzol, leptosol, arenosol, histosol, urban, and other). All models were fitted in R using the pscl package (Zeileis et al., 2008). No collinearity was found in the model residuals (generalized VIF [GVIF] <10), and spatial autocorrelation was low, with weak correlations between latitude and longitude and model residuals (±0.015).

A ZI model is capable of producing three types of predictions: (1) a prediction of abundance from the count component, (2) a prediction of abundance from the whole model, taking into account the processes generating the excess zeroes, and (3) a probability prediction (known as the zero prediction) that an observation is an excess zero. If all zeroes are true zeroes (i.e., there are no false absences), then the most accurate prediction of both observed and true abundance will be the second of these (abundance from the whole model) because the excess zeroes are the result of some underlying biological process. However, if a proportion of the excess zeroes results from sampling bias, then the count component prediction (hereafter known as the count prediction) may be a more accurate representation of the true species abundance, and the whole model prediction will partly reflect the processes underlying the sampling bias in the observed data. Therefore, the whole model prediction of abundance can provide insight into the sources of sampling bias in the model, whereas the count prediction provides estimates of abundance free from bias (Nolan, Gilbert, & Reader, 2021). Because the level of sampling bias in the ATI is unknown, both types of predictions could be informative and therefore were evaluated separately (Nolan, Gilbert, & Reader, 2021).

Model cross‐validation predictions (both count and whole model predictions of abundance) from the ZI models of ancient and veteran tree abundance for each 1‐km grid cell were created using 10‐fold cross‐validation, as described earlier. Predictions were evaluated using Spearman's rank correlation between predictions and raw abundance, root‐mean‐square log error (RMSLE), and training and test AUC. For each CV fold, training and test AUC were calculated by converting the abundance predictions from the Poisson and NB models into presence–absence predictions. The threshold chosen for this was allowed to vary across models and was the mean predicted abundance across all grid squares per model. The mean was chosen to create a more balanced data set of presences and absences to aid calculation of AUC.

### Field surveys and model verification

A set of 90 1‐km grid cells was selected across England for further independent model verification using field surveys. These squares comprised two groups: (1) 50 squares were selected completely at random so that there would be no additional sampling bias in the results and (2) 40 squares were selected based on model predictions to ensure that, despite the high proportion of squares containing no trees, there was good representation in the sample of squares with existing tree records in the ATI or predicted tree presences that could be verified. These 40 squares were selected using the highest performing ZI model abundance predictions (from the NB model). The ZI NB predictions were first categorized as being either low or high predicted abundance based on a threshold of the mean predicted probability that a square contained zero records (i.e., the mean zero prediction from the ZI model across each grid square). Then each square was categorized into one of four groups: (1) no ATI records and low predicted abundance, (2) no ATI records and high predicted abundance, (3) ATI records and low predicted abundance, and (4) ATI records and high predicted abundance. From each group 10 squares were randomly selected, resulting in the 40 ZI model squares.

Each of the 90 squares was assessed for accessibility using aerial maps and photography. If a square was deemed completely inaccessible (no roads or public rights of way present), then it was discarded and another square selected in the same manner (*n* = four out of 90). A survey form was created for each square containing details about location, what to record (number and location of ancient and veteran trees, date of survey, photographs), how to record any trees found on the form, possible car parking spaces for the recorder during the survey, and all roads and public rights of way. Recorders were also encouraged where possible to record species or genera of the trees found, although this was not included in the analysis in this study because of the relatively low number of individuals of each different species recorded (Appendix S1: Figure S2). This was likely because of the difficulty in identifying tree taxa when out of leaf during the late autumn/winter months, as well as problems classifying any trees that were recorded from a distance because of accessibility issues.

The aim of each survey was to cover each 1‐km grid as completely and thoroughly as possible, using multiple trips if necessary and binoculars to view areas from afar that were not accessible. To maximize the chances that every ancient and veteran tree in the square was found during the surveys, areas of interest were designated on each survey form to help the recorders avoid wasting their time sampling areas with a very low likelihood of ancient trees, for example, industrial parks, new housing estates, and open fields, determined using aerial photography and Ordnance Survey OpenStreetMaps. Only those areas deemed very unlikely to have any trees (or at least any ancient or veteran trees) were not covered under an area of interest. Therefore, we assumed that if all areas of interest had been surveyed with 100% coverage, then all ancient and veteran trees had been found. Each survey required the recorder to note the time spent surveying the whole square and each individual area of interest, as well as estimating the percentage from each area of interest that was covered during the survey. Any parts of the whole square that were not surveyed were the result of not being an area of interest, accessibility issues, or owing to a lack of time of the recorders.

Recorders comprised a range of volunteers from different sources, including the Ancient Tree Forum, Woodland Trust staff members, Woodland Trust ancient tree recorders, Woodland Trust ancient tree verifiers, and other independent volunteer ancient tree enthusiasts. Initially one square was assigned to each recorder, according to geographical proximity to their home, although some recorders completed several squares if no other recorder lived sufficiently close to that square. The recorders had no prior knowledge of any model predictions. Unfortunately, although squares were first assigned from March, due to extensive COVID‐19‐based travel restrictions at various points throughout 2020, many recorders assigned to squares were unable to complete them, and 39 out of 90 squares were completed by a total of 32 separate volunteers (Appendix S1: Figure S3). An additional 13 squares were completed by the authors, resulting in a total of 52 squares of the initial 90 (58%) being completed (Appendix S1: Figure S3). Although the authors had prior knowledge of the model predictions, care was taken wherever possible to carry out the surveys impartially. All surveys were carried out throughout the months of August to December, travel restrictions permitting, during daylight hours.

Three metrics were obtained from the field work results: (1) whether ancient or veteran trees were present or absent in each square (presence–absence), (2) raw abundance of ancient and veteran trees found in each square, and (3) estimated density of ancient and veteran trees per square in relation to survey effort of volunteer (number of ancient and veteran trees/estimated total area of the whole grid square surveyed in square meters). Presence–absence metrics were analyzed using AUC in relation to each of the model predictions of either habitat suitability (MaxEnt models) or abundance (ZI models). For this, the ZI model abundance predictions were converted to binary presence–absence form based on a threshold of the median prediction across all 90 grid squares. Median was chosen here instead of mean because several abundance predictions were extremely high and would therefore have skewed the mean, resulting in the majority of predictions being classed as absences. The raw abundance and density field work metrics were analyzed using Pearson's (*r*) and Spearman's (*r*_s_) correlation coefficients, and both coefficients were used to examine the effects of two potential outliers. AUC was selected based on the necessity of having a metric that could compare the predictions of abundance and habitat suitability; it is much more feasible to convert abundance to presence–absence rather than the other way around. This metric is not perfect and is likely to result in a loss of information from the ZI models. Using the correlations provides an alternative, albeit crude, method of direct assessment of the predictions against field verification results.

To calibrate the models and provide total estimates of ancient and veteran tree numbers across England, a linear regression model (Gaussian distribution, link = identity) was fitted for each set of model predictions for the 52 surveyed grid squares in relation to either raw tree abundance or tree density from the field surveys. Each of these linear regression models was then used to calibrate each model's predictions for all of the grid squares across England in order to provide predictions of abundance or tree density in each grid square. These estimates were then summed across all grid squares to predict the total number of ancient and veteran trees across England.

## RESULTS

### Model fitting and performance using internal model validation

All of the models were broadly supported by internal validation and allowed us to identify important predictors of spatial patterns in the abundance of ancient and veteran trees. A detailed comparison of how the models performed is given in what follows, but we focus first on describing the results of the ZI NB model, which performed well in internal validation and gave insights into the predictors of not only tree abundance (via the count component) but also sampling bias (via the excess zero component). Ancient and veteran tree abundance from the ZI NB model was found to be positively associated with higher altitudes, being closer to Tudor deer parks, commons (land owned collectively by many people with traditional shared grazing or harvesting rights), and National Trust sites (Table 3). Higher abundance was also associated with being farther away from towns and cities, having a greater coverage of forest and wood pasture but less coverage of orchard, and being associated with fewer minor roads (Table 3). Abundance also differed significantly across agricultural class, countryside type, land class, and soil type and was most likely to be highest on nonagricultural, freshwater, or broadleaved land classes and fluvisol soil type (Table 3).

**TABLE 3:** Model coefficients (±SE), *Z* values, and indication of the *p* value of significance are shown for negative binomial zero‐inflated model for both count and zero components. ﻿﻿﻿﻿﻿﻿﻿﻿﻿﻿

Model predictor	Count component		Zero component	
	Coefficient (±SE)	*Z* value	Coefficient (±SE)	*Z* value
Intercept	−3.276 (0.670)	−4.893***	−3.704 (1.330)	−2.785**
Agricultural class—agricultural	0.601 (0.279)	0.031*	−0.894 (0.359)	−2.490*
Agricultural class—nonagricultural	0.703 (0.283)	0.013*	−0.338 (0.368)	−0.918
Altitude	0.074 (0.033)	0.026*	0.147 (0.039)	3.809***
Type of countryside—ancient	0.036 (0.045)	0.431	−0.669 (0.060)	−11.20***
Type of countryside—highland	−0.328 (0.067)	−4.926***	−0.610 (0.091)	−6.672***
Type of countryside—Cornwall	0.088 (0.135)	0.514	1.413 (0.141)	10.02***
Landclass—broadleaved	2.349 (0.597)	3.937***	0.416 (1.031)	0.403
Landclass—heather/bog	1.529 (0.610)	2.509*	1.031 (1.031)	0.999
Landclass—saltwater	2.072 (0.753)	2.752**	1.664 (1.137)	1.465
Landclass—freshwater	2.783 (0.637)	4.368***	0.639 (1.066)	0.600
Landclass—coastal	1.268 (0.684)	1.855	1.654 (1.090)	1.517
Landclass—coniferous	2.100 (0.604)	3.477***	1.963 (1.031)	1.904
Landclass—urban	2.322 (0.596)	3.893***	1.109 (1.024)	1.083
Landclass—arable	1.991 (0.594)	3.355***	0.802 (1.019)	0.787
Landclass—grassland	2.152 (0.593)	3.627***	0.625 (1.019)	0.614
Soil type—luvisol	0.455 (0.123)	3.699***	0.692 (0.213)	3.246**
Soil type—cambisol	0.227 (0.122)	1.857	0.702 (0.212)	3.303***
Soil type—gleysol	0.310 (0.124)	2.498*	0.912 (0.215)	4.241***
Soil type—fluvisol	0.574 (0.158)	3.638***	1.242 (0.239)	5.207***
Soil type—podzol	0.360 (0.147)	2.449*	0.852 (0.253)	3.364***
Soil type—leptosol	0.406 (0.137)	2.953**	0.434 (0.228)	1.905
Soil type—arenosol	0.022 (0.157)	0.139	0.681 (0.262)	2.601**
Soil type—histosol	−0.573 (0.295)	−1.943	1.366 (0.350)	3.900***
Soil type—urban	0.266 (0.140)	1.894	1.200 (0.249)	4.812***
Tudor deer park	−0.130 (0.029)	−4.494***	0.500 (0.036)	13.92***
Moated site	−0.050 (0.034)	−1.477	−0.321 (0.037)	−8.639***
Historic forest	0.003 (0.022)	0.146	−0.252 (0.029)	−8.632***
Medieval deer park	−0.041 (0.021)	−1.955	0.080 (0.028)	2.874**
National Trust	−0.380 (0.021)	−17.71***	0.275 (0.028)	9.644***
Cities	0.120 (0.025)	4.856***	0.012 (0.032)	0.383
Towns	0.095 (0.028)	3.391***	−0.016 (0.034)	−0.486
Commons	−0.096 (0.017)	−5.545***	0.079 (0.024)	3.265**
Major roads	0.013 (0.023)	0.566	−0.050 (0.028)	−1.755
Cover of forest	0.226 (0.028)	8.177***	−0.312 (0.039)	−8.057***
Cover of ancient woodland	−0.009 (0.018)	−0.478	−0.475 (0.071)	−6.696***
Cover of orchard	−0.020 (0.010)	−1.990*	−0.778 (0.097)	−8.014***
Cover of wood pastures	0.374 (0.012)	31.93***	−18.99 (3.498)	−5.431***
Watercourse	0.030 (0.016)	1.883	−0.293 (0.024)	−12.08***
Minor roads	−0.117 (0.026)	−4.473***	−0.653 (0.050)	−13.13***
Log (θ)	−2.105 (0.019)	−113.1***	…	…

*** *p* < 0.001.

** *p* < 0.01.

* *p* < 0.05.

Potential predictors of sampling bias (inferred from the ZI NB zero component) suggest that the likelihood that a square is an excess zero (e.g., potentially unsampled) increase with increasing coverage of minor roads, wood pasture, orchard, ancient woodland, and forest (Table 3). Squares were also more likely to be excess zeroes if they were farther from watercourses, historic forests, and moated sites and were closer to commons, National Trust land, and medieval and Tudor deer parks. The likelihood that a square has not been sampled also increased if the square was at a lower altitude and covered certain land types, soil classes, and countryside types compared to others (Table 3). Interestingly, moated sites, historic forests, medieval deer parks, ancient woodland, and watercourses had a significant influence only in the zero component, suggesting they are stronger influences on sampling than on the true underlying ecology determining the tree distribution.

**FIGURE 2:**
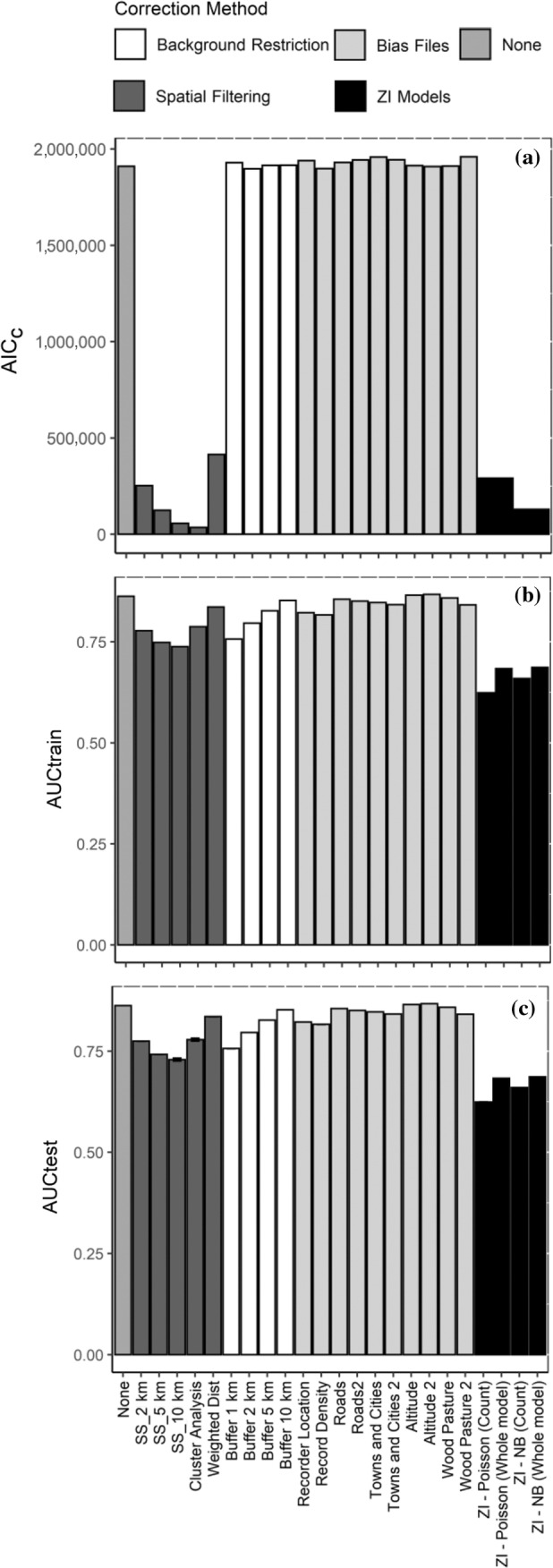
(a) Corrected Akaike information criterion (AIC_c_), (b) training area under curve (AUC), and (c) testing area under curve (AUC) for each species distribution model of ancient and veteran trees across England using four main types of bias correction method (spatial filtering, background restriction, bias files, and zero‐inflated [ZI] models) (see *Methods* for more information). Where the chosen bias source is also a model predictor, models were fitted with and without the predictor; models missing the predictor are indicated with “2.” The two (ZI) models were fitted using either a Poisson or a negative binomial (NB) distribution. Predictions of both abundance from the count component (“count predictions”) and whole model (“whole model predictions”) are shown. Error bars represent ± variance.

Internal model validation suggests that the highest performing bias correction method based on AIC_c_ was the cluster analysis spatial filtering technique, followed by systematic sampling at a 5‐ and 10‐km resolution (Figure 2a). All other spatial filtering methods also performed better than the model with no bias correction. Similarly, ZI models performed well compared to other methods, particularly when using a NB distribution. All other bias correction methods showed little difference compared to a model with no bias correction. The most effective bias file was record density, followed by altitude and wood pasture (Figure 2a), with the least effective being towns and cities. Nevertheless, the differences among all bias files were relatively small. There was also little difference between the background restriction methods, all of which performed relatively poorly.

When tested against the data used to build the models using AUC, there appeared to be little improvement in model predictive power when using any bias correction method in relation to the model with no bias correction (Figure 2b,c). Nevertheless, models fitted with bias files provided the best predictions overall based on both training and test AUC, particularly those using altitude, wood pasture, and roads. Background restriction using a 10‐km buffer was the best background manipulation method, and weighted distance was the best spatial filtering method. ZI models performed relatively poorly based on predictive power compared to all other models, although, as mentioned in the methods, this could have been because of the loss of information when converting abundance to presence–absence to calculate AUC.

As suspected, the ZI NB model provided a better fit to the data than the ZI Poisson model (Vuong AIC_c_ test: *Z* = −22.72, *p* < 0.001; NB AIC_c_ = 128,783.0, df = 80; Poisson AIC_c_ = 290,932.5, df = 81). Evaluation of model predictions using internal model validation showed support for the NB model having overall greater predictive power compared to the Poisson model (Figure 2b,c). Additionally, as well as the NB model outperforming the Poisson model, the whole model predictions showed stronger correlations to the raw data (Poisson *r*_s_ = 0.257 and NB *r*_s_ = 0.277) than the count predictions (Poisson *r*_s_ = 0.203 and NB *r*_s_ = 0.226), as well as lower error margins (whole model prediction RMSLE: Poisson—0.566, NB—0.583; count prediction RMSLE: Poisson—1.492, NB—0.706). This could be because the excess zeroes, in addition to being the result of sampling bias, are sometimes caused by ecological processes (e.g., biological aggregation), so excluding the zero component completely from the model predictions (as in the count prediction) might remove important biological information from the abundance prediction. Nevertheless, it could also be the result of other processes, for example, a poor choice of predictors of abundance or unexplained variation from human factors.

### Model validation using independent random field surveys

New independent surveys of 52 1‐km grid squares resulted in a total of 459 ancient and veteran trees being recorded (94 ancient and 365 veteran), 285 of which had not previously been recorded in the ATI. Before the surveys, only 15 of 52 squares had records of ancient or veteran trees, but this number was increased to 38 of 52 following the surveys. Seven squares received 100% survey coverage, and 32 squares (62%) had at least 50% of their area surveyed (Appendix S1: Figure S4). Accessibility was an issue for some squares, although only three squares received a survey coverage of <20%.

Calibrated predictions of the total number of ancient and veteran trees across England are very similar across all models, with around two million trees (1.7–2.1 million) predicted based on the estimated tree density (which accounts for estimated survey effort) from the field validation from all models (Table 4). This prediction ranges from 1,725,977 when using the spatial filtering technique, cluster analysis, to 2,088,979 when using the wood‐pasture bias file (so the range across all models is 363,002 trees). Predictions of the total number based on the raw abundance with no correction for survey effort are obviously lower and range from 826,052 with the wood‐pasture bias file to 1,120,545 (towns and cities bias file) (Table 4).

**TABLE 4:** Independent field evaluation of model predictions. Model predictions were evaluated against (a) field verification estimates of presence–absence (P–A) of ancient and veteran trees per square using area under the curve (AUC), (b) field estimates of raw tree abundance (total number of trees recorded per square) using Pearson's (*r*) and Spearman's (*r*_s_) correlation coefficient tests, and (c) field estimates of tree density (number of trees in relation to estimated percentage cover of each square) also using Pearson's and Spearman's correlation coefficient tests. See *Methods* for a detailed description of each bias correction method.

Model	P–A	Tree abundance			Tree density		
	AUC	*r*	*r*_S_	*T*	*r*	*r*_S_	*T*
None	0.613	0.589***	0.339*	911,842	0.490***	0.410**	1,867,480
ZI Poisson (count)	0.549	0.804***	0.190	873,219	0.667***	0.216	1,830,904
ZI Poisson (whole)	0.549	0.865***	0.286*	831,650	0.715***	0.329*	1,791,807
ZI NB (count)	0.600	0.929***	0.223	837,739	0.763***	0.275*	1,800,014
ZI NB (whole)	0.500	0.930***	0.270	831,096	0.765***	0.345*	1,792,819
SS 2 km	0.658*	0.239	0.431**	1,054,659	0.354**	0.534***	1,930,281
SS 5 km	0.664*	0.222	0.429**	1,045,737	0.421**	0.528***	1,862,409
SS 10 km	0.626	0.245	0.382**	1,051,987	0.391**	0.476***	1,912,788
Cluster analysis	0.618	0.642***	0.378**	842,714	0.666***	0.476***	1,725,977
Weighted distance	0.557	0.442***	0.224	1,008,806	0.366**	0.284*	1,961,867
Buffer 1 km	0.597	0.919***	0.185	1,091,502	0.749***	0.209	2,042,407
Buffer 2 km	0.614	0.852**	0.335*	991,488	0.712***	0.390**	1,943,748
Buffer 5 km	0.618	0.377**	0.309*	991,114	0.310*	0.362**	1,945,543
Buffer 10 km	0.602	0.400**	0.199	975,943	0.326*	0.259	1,932,274
Recorder density	0.586	0.463***	0.215	975,943	0.383**	0.251	1,894,780
Record density	0.443	0.584***	0.251	957,948	0.529***	0.319*	2,010,821
Towns and cities 2	0.470	0.156	0.080	1,097,494	0.135	0.110	2,045,136
Towns and cities	0.395	0.090	0.229	1,120,545	0.076	0.258	2,069,004
Roads 2	0.556	0.464***	0.179	1,008,015	0.382**	0.215	1,962,109
Roads	0.446	0.207	0.125	1,001,956	0.169	0.163	1,956,314
Altitude 2	0.591	0.627***	0.308*	964,732	0.532***	0.346*	2,049,180
Altitude	0.468	0.102	0.131	964,732	0.082	0.187	1,915,020
Wood pastures 2	0.621	0.869***	0.396**	1,141,381	0.755***	0.473***	2,088,979
Wood pastures	0.656*	0.708***	0.359**	826,052	0.582***	0.421**	1,788,310

*Note*: Values in bold represent those that are significant. Where indicated, significance levels are: *p < 0.05, **p < 0.01, ***p < 0.001. For each model the total predicted abundance of ancient and veteran trees (*T*) across England was calculated from a linear regression model between the model predictions and field verification data (both raw tree abundance and tree density) for the 52 surveyed squares.

Abbreviations: NB, negative binomial; SS, systematic sampling; ZI, zero‐inflated.

As with the ZI models, the most important predictor of ancient and veteran tree habitat suitability across all MaxEnt models was the cover of each square by wood pasture, which was especially true for the uncorrected model (Table 5), where it accounted for over 66% of variable importance. Other important predictors in the uncorrected model included National Trust land, cover of forest or ancient woodland, and soil type (Table 5). When using the optimum sampling bias correction method (systematic sampling—see following discussion for more information), wood‐pasture variable importance dropped significantly by almost 50%, although it was still the most important variable. Other big changes included an increase in permutation importance of the type of countryside and the distance to a Tudor deer park, both by 11% (Table 5). The most important predictors of ancient and veteran trees from the systematic sampling model were therefore similar to those of the ZI models and included wood‐pasture cover, cover of forest, distance to a Tudor deer park, type of countryside, and the presence of minor roads.

**TABLE 5:** Permutation importance of each of the maximum entropy (MaxEnt) distribution model predictors shown for model with no bias correction compared to overall best performing bias‐corrected model using systematic sampling (SS) at 2‐km resolution. The percentage change in permutation importance between the two models is also shown, with positive values representing variables that become more important when bias is corrected for and negative values are less important.

Predictor	Permutation importance		Percentage change
	No correction	SS (2 km)	
Agricultural class	0.209	1.230	1.021
Altitude	0.528	1.866	1.338
Type of countryside	0.193	11.86	11.667
Land class	1.734	1.929	0.195
Soil type	5.473	7.526	2.053
Tudor deer park	2.196	13.47	11.274
Moated site	0.000	1.249	1.249
Historic forest	0.010	3.893	3.883
Medieval deer park	0.703	0.143	−0.56
National Trust	7.947	6.560	−1.387
Cities	0.297	0.927	0.63
Towns	0.000	0.640	0.64
Commons	0.112	0.595	0.483
Major roads	0.007	0.506	0.499
Cover of forest	6.997	14.88	7.883
Cover of ancient woodland	5.672	1.856	−3.816
Cover of orchard	0.010	0.246	0.236
Cover of wood pastures	66.20	18.83	−47.37
Watercourse	0.801	3.556	2.755
Minor roads	0.909	8.247	7.338

**FIGURE 3:**
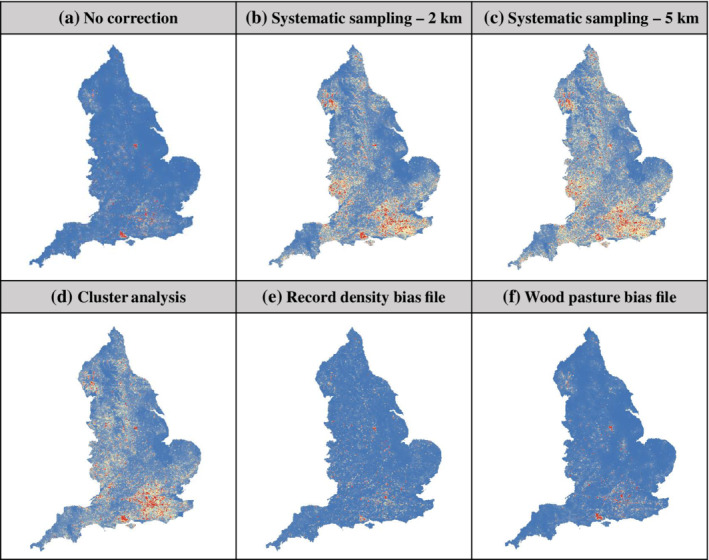
Predicted distribution maps of habitat suitability for ancient and veteran trees across England from (a) a model with no bias correction and some of the highest performing maximum entropy (MaxEnt) bias correction methods, (b, c) systematic sampling using grids of 2‐ and 5‐km resolution, (d) cluster analysis, (e) record density bias file, and (f) wood‐pasture bias file. Habitat suitability ranges from low suitability (blue) to high suitability (red).

**FIGURE 4:**
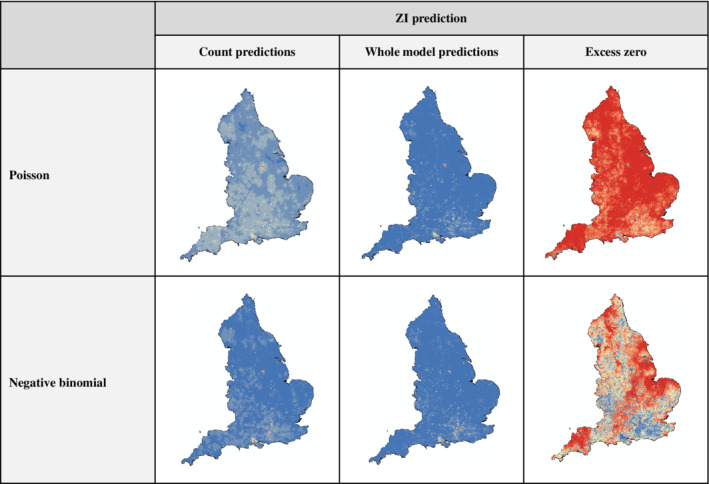
Predicted maps of abundance of ancient and veteran trees across England from Poisson and negative binomial (NB) zero‐inflated (ZI) models. Three types of predictions are shown: (1) count prediction only from count component of ZI model, (2) whole model prediction from whole ZI model, and (3) excess zero prediction, which represents probability that an observation is likely to be an excess zero (i.e., a “false absence”). Red areas in predicted abundance maps represent areas of high abundance, whereas in zero‐probability maps they represent places where there is likely to be undersampling.

Prediction maps of ancient and veteran tree distributions from models using bias correction showed substantial differences compared to the uncorrected model (Figure 3, see Appendix S1: Figures S6 and S7 for maps from all models), with much more variation in habitat suitability across England when using systematic sampling (Figure 3). The systematic sampling model prediction maps at scales of 2 and 5 km suggest that there are more areas with high suitability, especially in the southeast of England, the Lake District, and Herefordshire. In contrast, the bias file models using record density or wood‐pasture habitat suggest there are relatively few areas of high suitability, many of which are actually wood pastures (Figure 3). Prediction maps of abundance from the ZI models are displayed in Figure 4 and show some areas of high suitability, particularly around London and the New Forest National Park in the south. Maps of the zero predictions from the ZI models provide interesting insight into areas with high numbers of excess zeroes, where trees are likely to have been particularly under‐recorded. These maps suggest under‐recording in much of Cornwall and Devon, Norfolk, and other counties in the East of England and in parts of Northumberland.

Many of the bias‐corrected models produced predictions that strongly correlated with the field estimates of ancient and veteran tree abundance or tree density, and bias correction substantially improved the predictive power of the distribution models compared to the uncorrected model (Table 4). However, the evaluation of the performance of each model when predicting the raw abundance or density of ancient and veteran trees depended heavily on whether the raw values (Pearson correlation coefficients) or ranked values (Spearman correlation coefficients) were used. This discrepancy was caused by two outlier squares with extremely high predictions of abundance that were likely inflating the accuracy of the raw predictive power of some of the models (e.g., ZI) when evaluated with Pearson's correlation (Appendix S1: Figure S5).

Field estimates of both raw tree abundance and density based on the Spearman ranked correlations provided good support for the spatial filtering bias correction technique, systematic sampling, and showed significant, strong correlations with model predictions, particularly at 2‐ and 5‐km resolutions (Table 4). The only other methods that increased model predictive power relative to the uncorrected model were the cluster analysis spatial filtering technique and the wood‐pasture bias file (Table 4). When evaluated using estimates of survey effort (i.e., against tree density) rather than with the raw abundance of trees per grid square, all these techniques produced predictions with stronger correlations to the field estimates, and the best bias correction was still systematic sampling at either a 2‐ or 5‐km resolution, although using the wood‐pasture habitat as a bias file also produced good results (Table 4).

When considering the raw Pearson correlation coefficients, the ZI models performed much better in comparison with the uncorrected model, with very strong correlations between field estimates of both abundance and density and model predictions (Table 4). This is especially true of the ZI NB model, and, based purely on this evaluation metric, the ZI NB appeared to be the best method of all to deal with sampling bias. However, because of the outlier grid squares (Appendix S1: Figure S5), Spearman correlations are likely a better measure of performance, but it is interesting to see the high performance of the ZI models at correctly predicting squares with very high abundances of trees (Appendix S1: Figure S5).

## DISCUSSION

In this study, we have presented a rare empirical test of the ability of models fitted using a large citizen‐science species database to provide an unbiased prediction of the distribution of ancient and veteran trees across a large geographic area. Our results using robust independent field verification methods showed that there are indeed many undiscovered ancient and veteran trees across England and that only a small proportion of the ancient and veteran tree population has been mapped. By evaluating and selecting the best bias correction methods to apply to our distribution models, we can produce accurate predictive maps of the locations of these previously unknown trees to inform future targeted surveying and conservation plans for these valuable components of terrestrial biodiversity.

It has long been suspected that there are many unrecorded ancient and veteran trees across England with great ecological importance in terms of their dead‐wood habitats and associations with saproxylic species (Butler et al., 2002; Fay, 2004; Read, 2000). Our study provides strong support for the existence of these trees and demonstrates that taking a comprehensive, targeted survey approach is an excellent method for this purpose. Our field surveys covered a very small percentage of the area of England (0.04%), yet they increased the number of known ancient and veteran trees by a total of 285 records, more than a 100% increase in the number of trees recorded in the ATI in these locations before the surveys. From these surveys alone we have seen clear large gaps in our knowledge of the current distributions of these trees, suggesting that many of them may remain unaccounted for in current strategies for protection, ecological monitoring, and management. This is true despite the fact that in the UK such trees are much better recorded at the level of the individual than they are in most other parts of the world. Based on this study, future surveys following similar, partially randomized protocols, or ideally and if possible completely randomized protocols that aim for 100% survey coverage of a targeted specific area would greatly improve the discovery rate of the trees across the landscape.

Based purely on the raw abundance of trees recorded during the surveys, total estimates of ancient and veteran trees across England were around one million, more than five times the number currently in the ATI. However, when estimates of sampling effort for each square were factored in, to account for the parts of each square that were inaccessible in the field survey, the estimated total based on tree density is around two million trees for several models. This is the first study to provide quantitative nationwide estimates of the true number of ancient and veteran trees; our previous research focused purely on wood pastures in England, which cover an area of ~2780 km^2^, and the study predicted around 100,000 such trees just in this habitat (Nolan, Reader, et al., 2021). Other estimates have guessed figures close to nine million ancient or veteran trees across the whole UK (Fay, 2004), so our estimates do not seem wildly inflated. Nevertheless, our results suggest that there is much work to do to find these trees and add them to the ATI.

Field validation with independent, unbiased sampling is probably the gold standard when evaluating the performance of distribution models and predictive maps, and yet it is rarely used (Getz et al., 2018). Other model validation methods exist, for example, the use of aerial or historical maps (Nolan, Reader, et al., 2021), but most commonly model performance is assessed using methods of internal validation: Often the retention of a portion of the data to test the models or a cross‐validation approach is considered sufficient to validate models and make accurate predictions (Fielding & Bell, 1997), with AUC the most common evaluation statistic used for this. However, measuring model accuracy using AUC and cross‐validation has been criticized because it is likely to inflate perceptions of model performance owing to spatial autocorrelation in the species data (Lobo et al., 2008; Peterson et al., 2008). Additionally, any data retained to test the model from a biased species data set will suffer the same bias as the data used to fit the model, thereby giving false confidence that significant predictors of species occurrence are predicting the underlying ecology, when they are actually predictors of sampling effort. Therefore, to evaluate models fully and to assess the utility of different sampling bias correction methods, it is important to use unbiased field data where possible.

Because they were collected under standardized conditions, and recorders were blind to model predictions, the new field data with which we sought to validate our models were both free from many of the sources of bias likely to be present in the ATI data set (see *Introduction*) and independent in the sense that model predictions should not have influenced how abundance was estimated for the sites targeted. However, our choice of sampling strategy was not entirely random: We chose a proportion of squares to survey based on the model predictions to ensure some squares would contain trees and, thus, increase the chances that our relatively low power field test would detect a signal of the relationship between model predictions and the ground truth, against the inevitable background noise. This nonrandom element in our sampling means that our field data were not entirely free from sampling bias and that the value of the inferences made from them could be affected by the issues known to be associated with “preferential sampling” in studies of this kind (Diggle et al., 2010). Nevertheless, we believe that the very different and highly standardized method of data collection we employed, relative to that used to create the original data set, provides a much more robust validation of the conclusions we reached than would have been possible with internal validation alone. Thus, we believe our maps are likely to be biologically informative and robust against the obvious sampling bias in the ATI, something that relatively few studies can claim. Alongside fine‐tuning modeling procedures and understanding ecological systems, the feasibility of collecting additional data for model validation should always be an important consideration of an ecological study.

Spatial filtering, especially the systematic sampling technique, proved to be one of the most effective bias correction methods overall based on both internal validation using AIC_c_ and field validation. This method is known to be particularly useful for wide‐ranging, heavily sampled species and has been shown to reduce both type I and type II errors (Kramer‐Schadt et al., 2013). However, it is often limited by sample size because reducing the number of occurrence records can result in poor model predictions. Furthermore, the best choice of spatial filter may differ depending on environment; for example, Boria et al. (2014) suggested that mountain regions need smaller spatial filters than other areas. There is also the risk of reducing clustering in areas that truly represent high ecological value for a species (Fourcade et al., 2014). Nevertheless, the large number of records in the ATI, as well as the large range of the trees across the UK, means spatial filtering is likely to be highly effective for this database. A similar study using spatial filtering with large species databases also reported good results compared to independent field data (Law et al., 2017), and the researchers concluded that their models were suitable for application to practical management scenarios. We believe that our similarly high‐performing, independently validated models are also suitable for management applications and could provide valuable insight into the areas most suitable for immediate practical ancient and veteran tree conservation measures.

It is notable that field validation often ranked models differently compared to internal model validation; based purely on internal model evaluation, we would have inferred that the best bias correction method was the cluster analysis spatial filtering technique, followed by the ZI models, both of which performed less well when evaluated against the field data using AUC or Spearman rank correlations. The performance of the bias files also differed greatly between internal and field validation, although wood‐pasture habitat performed well using both methods. Wood pastures have strong connections to ancient and veteran trees and are the most studied of the habitats in which these organisms are found (Farjon, 2017; Hartel et al., 2018; Rackham, 1994). Additionally, many wood pastures in the UK form part or the whole of a site of interest from a tourism or aesthetic point of view, for example, National Trust sites or public parkland (Lonsdale, 2013; Rackham, 1994). Therefore, it is no surprise that wood‐pasture spatial distributions exert strong influences on recorded ancient and veteran tree distributions, via effects on both ecology and sampling effort: Both the count prediction of abundance and the probability of a grid square being sampled from the ZI models were predicted to be higher in grid squares with greater coverage of wood pasture. In the bias‐corrected MaxEnt models, wood‐pasture importance as a predictor did decrease significantly compared to the uncorrected model, suggesting it has a significant influence on sampling bias in the ATI, yet it remained the most important predictor overall. This explains why in all the predicted distribution maps, even when sampling bias was corrected for, many grid squares contained wood pastures that had very high suitability for ancient and veteran trees. It is therefore of high importance to protect habitats like wood pastures or National Trust sites for the conservation of ancient and veteran trees, and studies like ours can provide the data (e.g., predictions of high numbers in these areas) in support of changes to policy and conservation measures concerning these habitats that will benefit these trees.

Background manipulation methods also performed differently between internal model and field validation. They were relatively good at predicting raw tree abundance found during the field surveys, especially in squares with high numbers of trees, but not so good at predicting tree density (accounting for survey effort estimates) or producing models that fitted well to the original data. Although there has been some success with this method in other studies (Phillips et al., 2009), it was previously considered to perform worse than other methods (Fourcade et al., 2014), possibly because background points were restricted to too narrow an area, reducing model accuracy (Thuiller et al., 2004). Understanding the optimum background area size and considering both the species range and the extent of sample bias are likely to be case specific and should be considered before using this method for bias correction.

The performance of ZI models varied the most across validation methods; internal model evaluation showed that ZI models provided a very good fit to the raw data (low AIC_c_ values) but low predictive power (poorer AUC performance), whereas field validation suggested that the models were very well suited to predicting raw abundance or density, especially of outlier observations where abundance was high, but poor at predicting presence–absence and ranked abundance and density. It is important to note that variation in model performance will likely differ across study species, geographic area, and sampling protocols. Therefore, the best models presented here for ancient and veteran trees will not always be the best choices in other scenarios, and we advise researchers to design and test their own hypotheses about sampling bias patterns in their data. However, we do believe that ZI models are highly suitable for modeling sampling bias and should be considered as candidate methods in future studies because of their benefits compared to other models. For example, these were the only models to provide some independent insight into potential causes of bias in the original data by examining potential causes of excess zeroes (Nolan, Gilbert, & Reader, 2021). The ZI models used showed that many predictors had some influence on the proportion of excess zeroes in the ATI data, the majority of which are likely to influence both the ecology of the trees and the likelihood of them being sampled, including altitude, type of land or soil, distance to roads and watercourses, historic land use, and cover of forests, woods and wood pasture. Nevertheless, it is likely that predictors that also influenced the count component of the ZI model, for example altitude, have a greater influence on the ecology, whereas those influencing only the zero component, such as distance from a watercourse, are more likely reliable indicators of sampling effort. The high number of predictors potentially influencing both the tree ecology and sampling processes is likely the reason why the whole model predictions were better overall than the count predictions: A proportion of the excess zeroes in the ZI zero component are probably biological zeroes rather than being caused by undersampling. Removing the influences of these processes from the model predictions (which is what the count predictions do) would therefore remove meaningful biological information from the overall prediction maps.

Another major benefit of ZI models is that they can be used to generate distribution maps of the predicted excess zeroes, providing insight into areas that may have been under‐ or oversampled, thereby helping those planning future sampling and conservation efforts. In our study, Cornwall and Devon counties were, for example, predicted to have high numbers of excess zeroes and are therefore good candidates for extra targeted surveys. Although ZI models were previously used to fit SDMs (Bouyer et al., 2015; Lyashevska et al., 2016), this is the first time they were successfully applied to identify causes of and to correct for sampling bias, and our results highlight their potential advantages over other more conventional methods of sampling bias correction. We believe ZI models have strong potential in the fields of ecological modeling and practical conservation.

Our results first and foremost provide a robust prediction of ancient and veteran tree distributions across England that can be used for conservation planning and decision‐making. Until now, there has been no real measure of the landscape‐scale value of this habitat and how it interconnects. Our work shows the overall collective value of this irreplaceable natural resource and should frame the debate for further serious discussion about what level of effort will be required to map, monitor, and manage ancient and veteran trees in the future. In addition, despite the difficulties presented by a global pandemic, our study demonstrates how citizen scientists can be mobilized to conduct independent field validation of models built from large publicly accessible databases, increasing confidence in and the utility of model predictions. Our results also underline the impact of sampling bias in citizen‐derived data sets on the effectiveness of ecological models in conservation. Correcting for sampling bias is essential for preventing incorrect inferences from distribution models influencing practical conservation decisions.

## AUTHOR CONTRIBUTIONS

Victoria Nolan conceived of the presented idea and performed the modeling, data analysis, and some field work. Tom Reader and Francis Gilbert verified the methods and provided input to the supervision of the project. Tom Reed assisted especially in establishing the methodology for the field work and handling the volunteer recruitment and data collection. The initial draft was written by Victoria Nolan with final draft input from all authors.

## CONFLICT OF INTEREST

There are no conflicts of interest to report in relation to this manuscript.

## Supporting information


Appendix S1

Appendix S2

## Data Availability

The main data set utilized for this research was the Ancient Tree Inventory, which can be requested for research purposes from Woodland Trust (https://ati.woodlandtrust.org.uk/). The exact records used here from this data set are described in *Methods: Study species and environmental predictors*, where all query information is provided. All other data sets used in this manuscript can be found in Table 1. R code and other data for the analysis (Nolan et al., 2022) are available in Figshare at https://doi.org/10.6084/m9.figshare.14113172.
