# Supplementary material for: Distribution models calibrated with independent field data predict two million ancient and veteran trees in England
Source: Ecol Appl. 2022 Aug 9;32(8):e2695. doi: 10.1002/eap.2695 (PMC10078183; doi:10.1002/eap.2695)
Supplement: Supplementary file 1 — Appendix S1 [file EAP-32-0-s001.pdf]

Appendix S1

Distribution models calibrated with independent field data predict two million ancient and veteran trees in England

Victoria Nolan, Francis Gilbert, Tom Reed and Tom Reader

Ecological Applications

Additional tables and figures

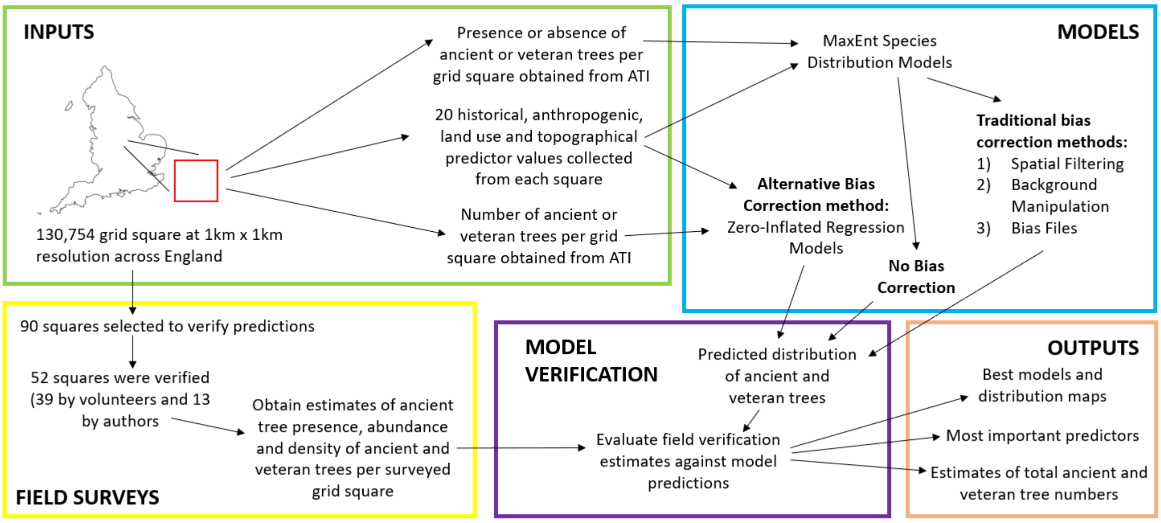

Fig. S1 *Flow chart summarising the methodology and modelling processing employed to generate predictions of ancient and veteran tree abundance, based on data from the Ancient Tree Index (ATI). Processes include selection of model inputs, the models fitted and bias correction methods, field surveys, model prediction verification and final outputs.*

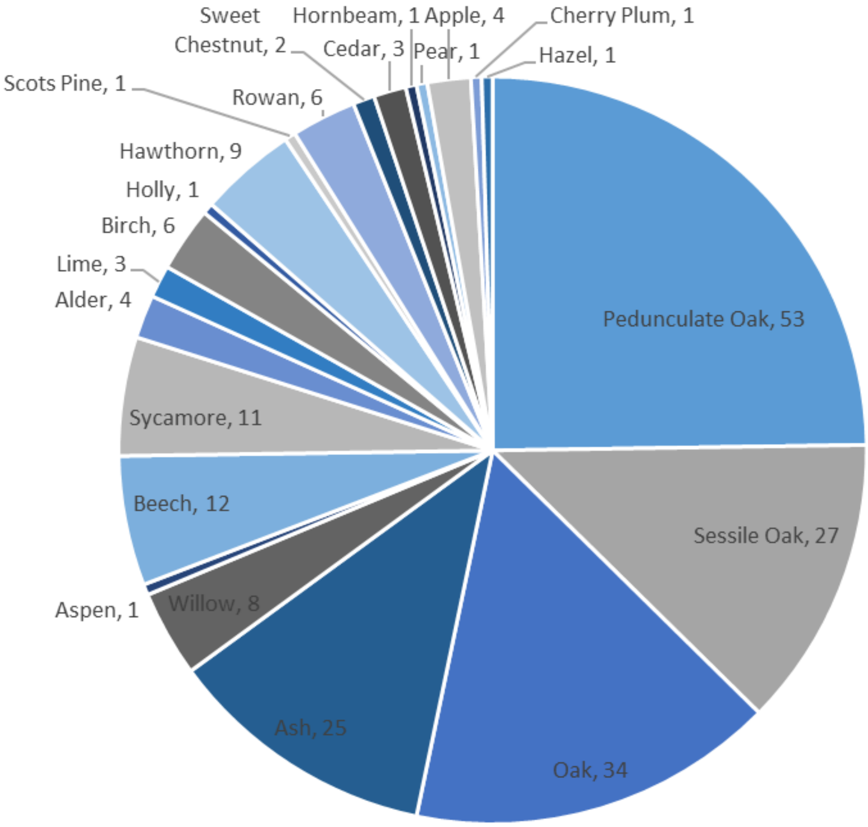

Fig. S2 *Number of each species/ genera of tree that was able to be identified out of the 52 surveyed grid squares from the field verification work.*

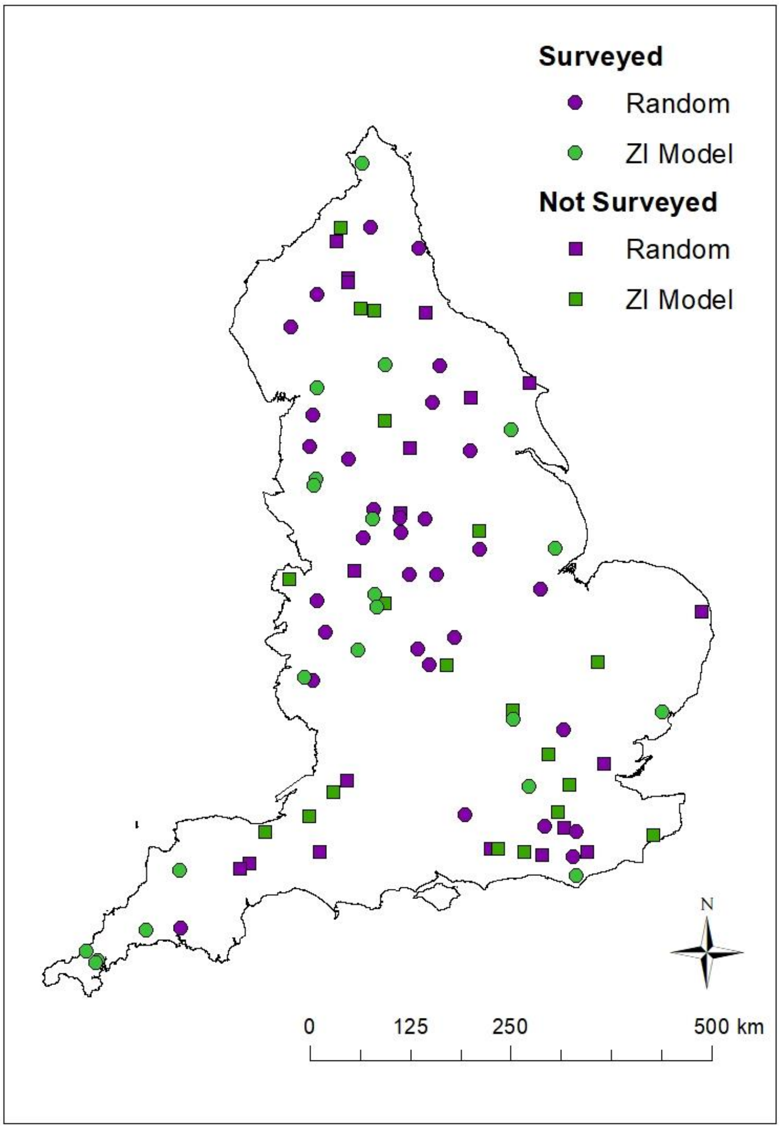

Fig. S3 *Centroid locations of each of the 90 1-km grid squares selected for field verification. 50 squares (purple) were selected at random across England and 40 squares (green) were selected based on the model predictions from the zero-inflated (ZI) models. Squares that were actually surveyed for the field verification are indicated as circles, whereas those that were not able to be surveyed due to travel restrictions are indicated as squares.*

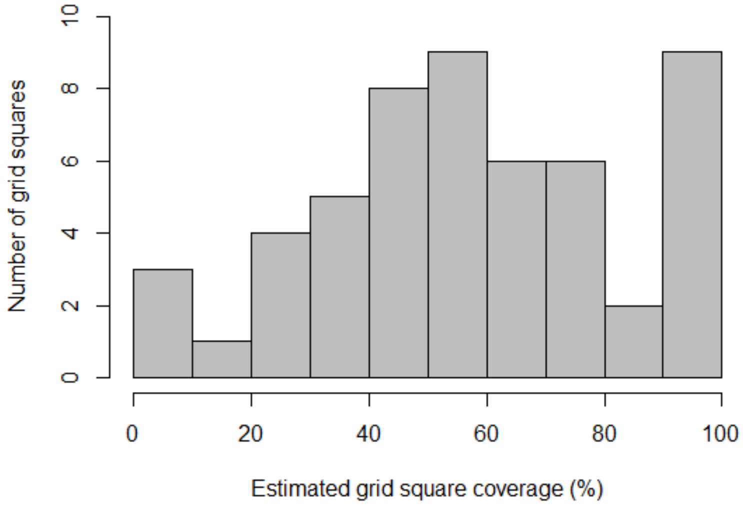

Fig. S4 *Histogram of the estimated percentage coverage of each grid square during the field surveys. Percentage coverage was estimated by totalling the area covered from each 'area of interest' and any other areas that the recorders were able to survey.*

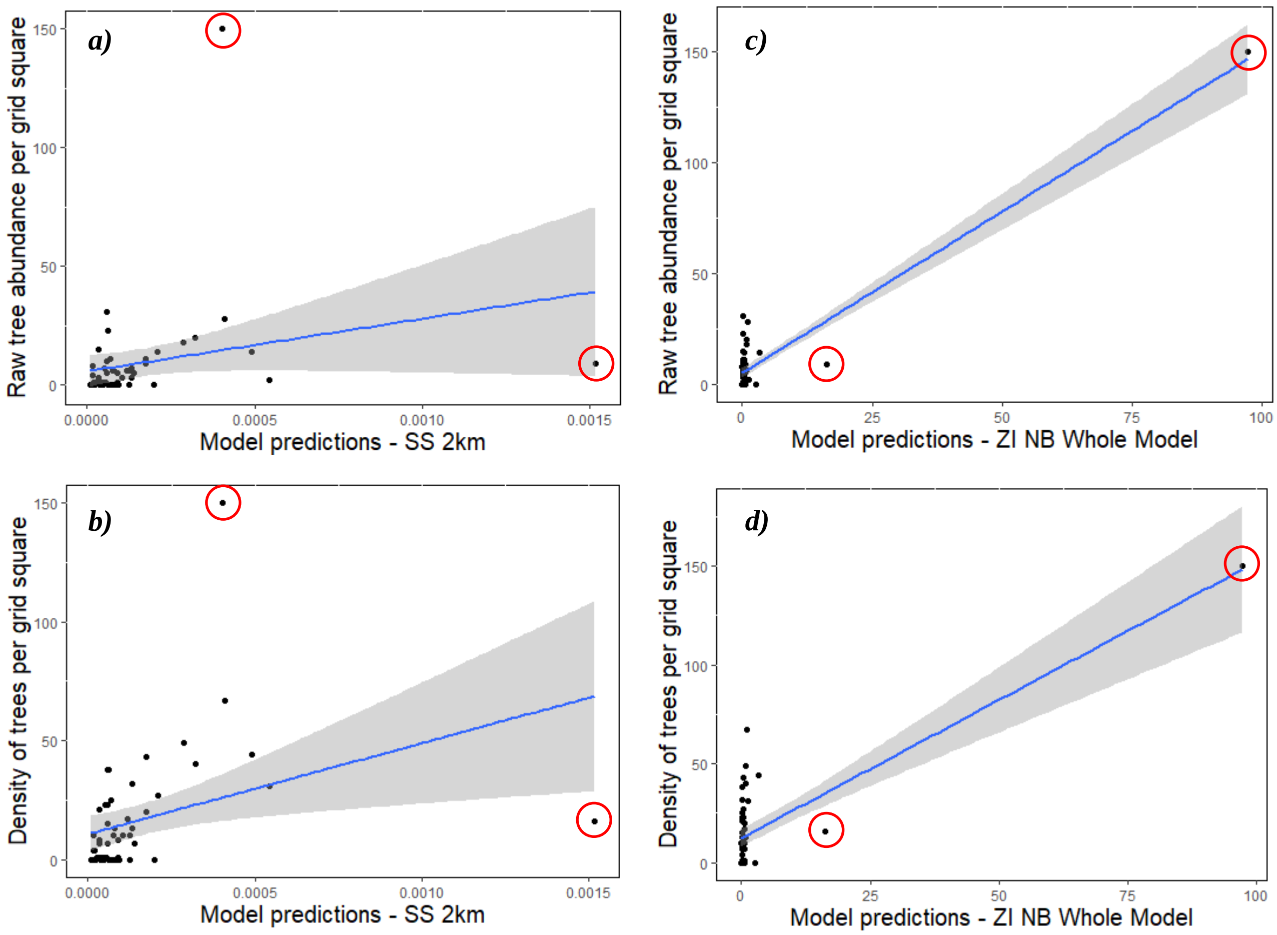

Fig. S5 *Scatterplots of model predictions (±SE) from the ancient and veteran tree distribution model fitted using systematic sampling (SS) at a 2-km resolution (i.e. the best overall performing Maximum Entropy (MaxEnt) model) or the Zero-Inflated (ZI) negative binomial (NB) model in relation to estimates from the 52 surveyed grid squares of a & c) the raw field verification abundance estimates and b & d) estimates of density of trees (including estimates of survey effort). Two grid squares (circled in red) are deemed to be outliers.*

Fig. S6 *Predicted maps of ancient and veteran tree distributions (or abundance from the ZI models) from each model with and without a bias correction method. Predicted areas of high suitability are represented in red, whereas predicted areas of low suitability are represented in blue. Map predictions from each model are not shown to the same colour scale.*

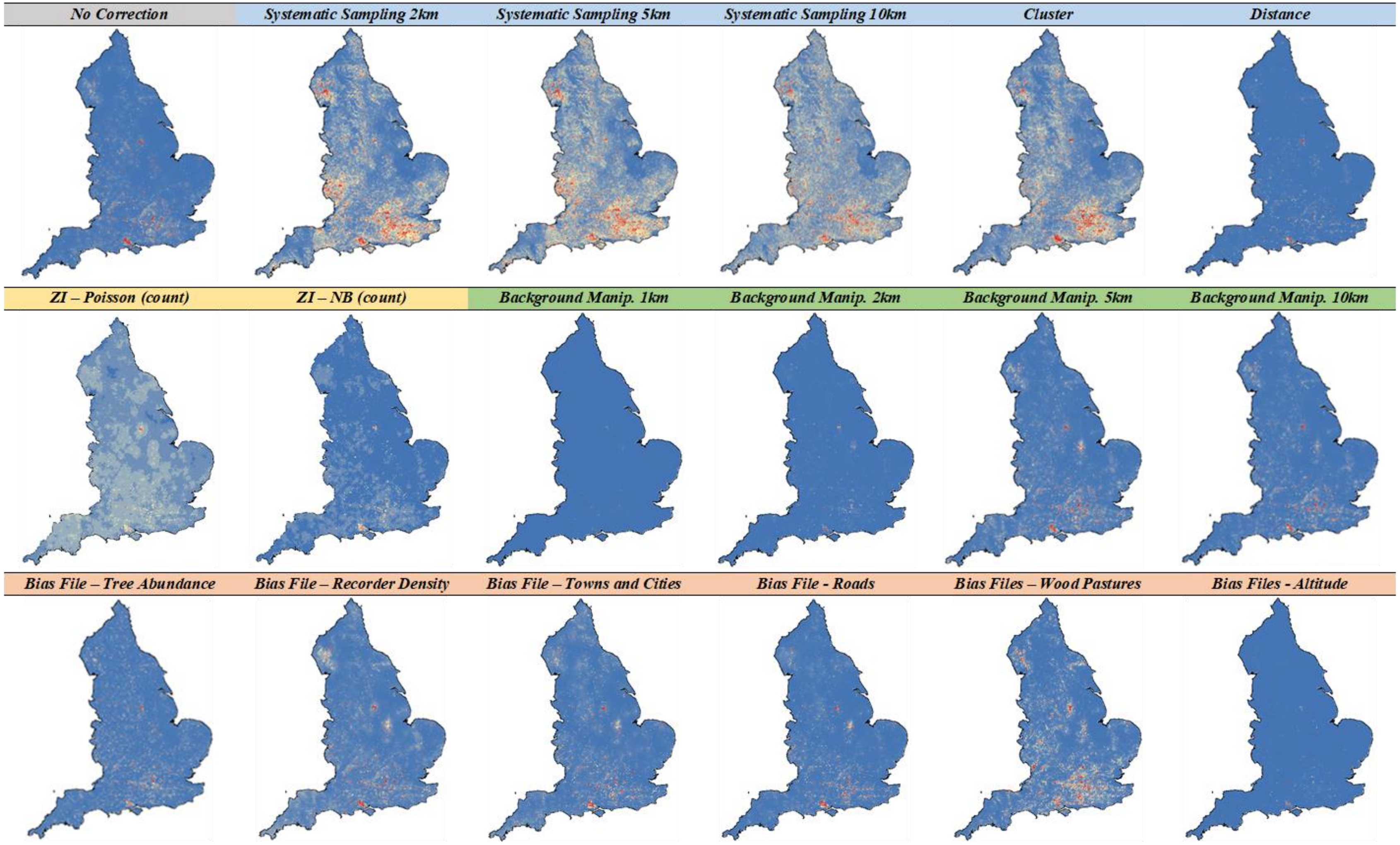

Fig. S7 *Calculated differences between predicted maps of ancient and veteran tree distributions with no correction and each predicted distribution map from a model using a bias correction method. Abundance predictions from the ZI models were first scaled between 0 and 1 before calculating their difference from the probability predictions from the model with no bias correction, Blue squares represent areas that are predicted to be less suitable following the application of bias correction. Difference maps from each model are not shown to the same colour scale.*

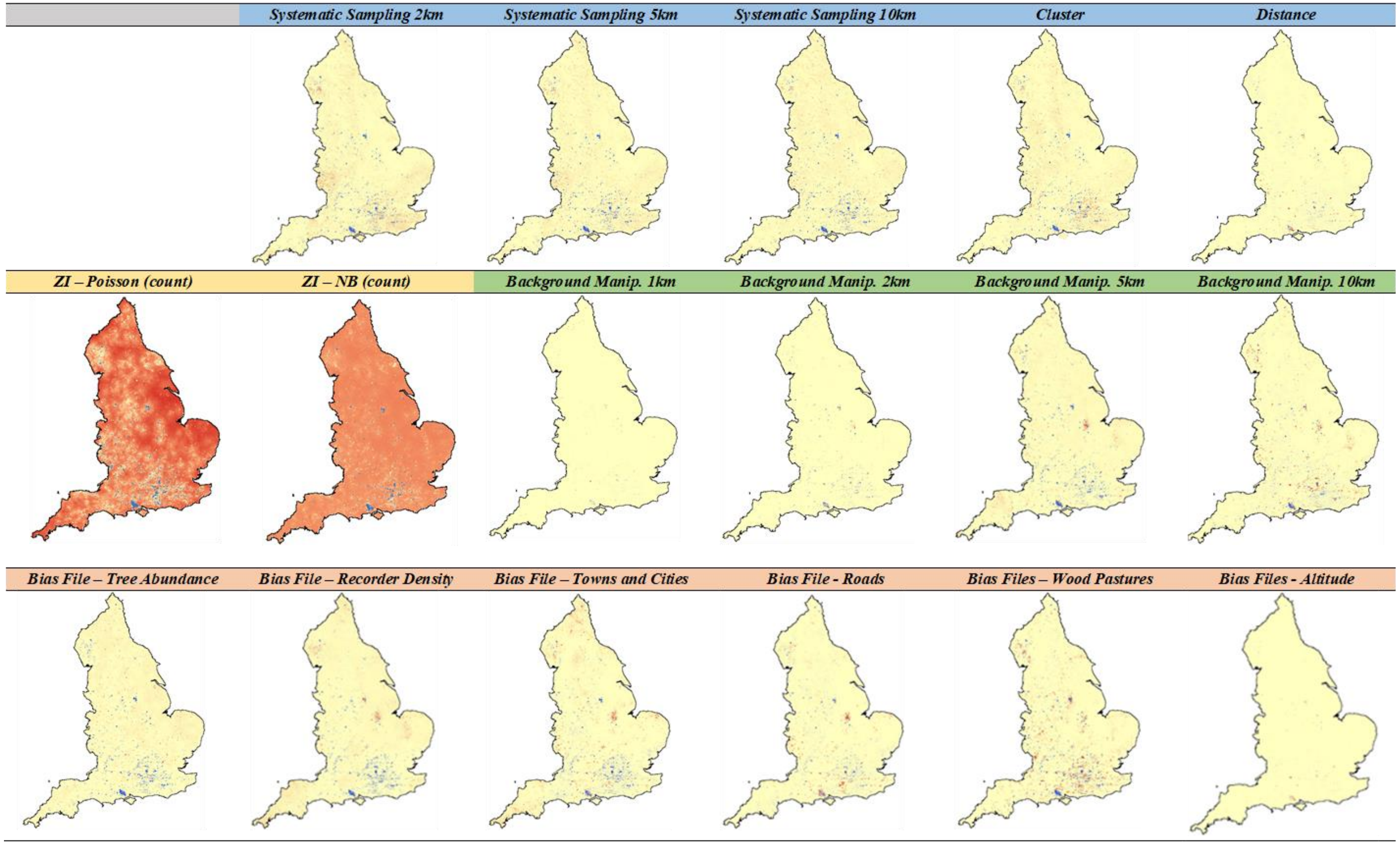

Table S1 *The Ancient Tree Inventory (ATI) star rating system with the reason for the rating.*

Rating	Reason for rating
**5**	Recorded and verified by WT verifiers on site
**4**	Verified by a WT verifier or Quality Assured records but not verified on site
**3**	Verified by volunteers of another organisation
**2**	Data that has proved unreliable and unverified
**1**	Unverified but with potential of being 5 star

Table S2 *Guide to the grouped land classes. Adapted from 'Land Cover Map 2015 – Dataset documentation V.1.0 (CEH, 2017)'.*

Category	Habitats Included
**Broadleaf woodland**	Broadleaved, mixed and yew woodland
**Coniferous woodland**	Coniferous woodland
**Arable**	Arable and horticulture
**Improved grassland**	Improved grassland
**Semi-natural grassland**	Neutral, calcareous and acid grassland. Fen, marsh and swamp.
**Mountain, heath, bog**	Dwarf shrub heath (heather and heather grassland), bog and inland rock
**Saltwater**	Saltwater
**Freshwater**	Freshwater
**Coastal**	Supra-littoral and littoral rock, supra-littoral and littoral sediment, saltmarsh
**Built-up areas/ gardens**	Urban and suburban
**Other**	Other habitats not included in other categories

Table S3 *Guide to the WRB Reference Soil Groups (RSG) used to group the full soil codes. Adapted from the 'World reference base for soil resources 2014: International soil classification system for naming soils and creating legends for soil maps (2015)'.*

Characteristic	RSG
**Soils with thick organic layers**	Histosols
**Soils with strong human influence (e.g. intensive agriculture, containing artefacts)**	Anthrosols, Technosols
**Soils with limitations to Root Growth (e.g. permafrost, high concentration of soluble salts or Na, thin soil, alternate wet-dry conditions)**	Cryosols, Leptosols, Solonetz, Vertisols, Solonchaks
**Soils distinguished by Fe/Al Chemistry (e.g. stagnating water, presence of oxides or humus, accumulation of Fe etc.)**	Gleysols, Andosols, Podzols, Plinthosols, Nitisols, Ferralsols, Planosols, Stagnosols
**Accumulation of organic matter in mineral topsoil (e.g. dark topsoil, secondary carbonates etc.)**	Chernozems, Kastanozems, Phaeozems, Umbrisols
**Accumulation of moderately soluble salts or non-saline substances. (e.g. accumulation of secondary silica or carbonates)**	Durisols, Gypsisols, Calcisols
**Soils with clay-enriched subsoil**	Retisols, Acrisols, Lixisols, Alisols, Luvisols
**Soils with little or no profile differentiation (e.g. moderately developed, sandy, marine or sediments)**	Cambisols, Arenosols, Fluvisols, Regosols

Table S4 *Guide to the grouped agricultural classes. Adapted from 'Agricultural Land Classification of England and Wales - Revised guidelines and criteria for grading the quality of agricultural land' (Ministry of Agriculture, Fisheries and Food, 1988).*

Agricultural Class	Broad Description	Group
**Grade 1**	Excellent quality agricultural land	Grade 1
**Grade 2**	Very good quality agricultural land	Grade 2
**Grade 3a**	Good quality agricultural land	Grade 3
**Grade 3b**	Moderate quality agricultural land	Grade 3
**Grade 4**	Poor quality agricultural land	Grade 4
**Grade 5**	Very poor quality agricultural land	Grade 5
**Urban**	Housing, industry, commercial, education, transport etc.	Urban
**Non-agricultural**	Golf courses, parkland, sports fields, allotments etc.	Non- Agricultural
**Woodland**	Commercial and non-commercial woodland	Other
**Agricultural buildings**	Permanent agricultural buildings, glasshouses etc.	Other
**Land not surveyed**	Agricultural land that has not been surveyed	Other
**Open water**	Lakes, ponds and rivers	Rivers

Table S5 *Guide to the historic types of countryside, as defined in Rackham, 1976 - Trees and Woodland in the British Landscape.*

Countryside Type	Broad Description
**Ancient**	Lowland countryside. Hedged and walled landscape that can be traced back often to even the Bronze age. Fields are irregular and of varied origin with varied and thick hedgerows, hamlets, medieval farms, pollards and many ancient trees.
**Planned**	Lowland countryside. Regular fields, straight roads and small woods, derived following the Enclosure Acts in the 18th and 19th centuries. Features exposed buildings, thin hawthorn hedgerows, few roads and big villages. Medieval woods and ancient trees remain in places where they were failed to be destroyed after the enclosures.
**Highland (including highland in Cornwall)**	Coverage of moors, dales and mountains. Ancient woods are generally composed of Oak (*Quercus* spp.) and management declined earlier in these areas than in the lowland ancient or planned countryside.
