# Supplementary material for: Distribution models calibrated with independent field data predict two million ancient and veteran trees in England
Source: Ecol Appl. 2022 Aug 9;32(8):e2695. doi: 10.1002/eap.2695 (PMC10078183; doi:10.1002/eap.2695)
Supplement: Supplementary file 2 — Appendix S2 [file EAP-32-0-s002.pdf]

Appendix S2

Distribution models calibrated with independent field data predict two million ancient and veteran trees in England

Victoria Nolan, Francis Gilbert, Tom Reed and Tom Reader

Ecological Applications

Species distribution model parameter tuning and evaluation of alternative methods of splitting of training and test data using Maximum Entropy modelling.

Section S1: Introduction

When fitting any species distribution model (SDM) the choice of parameters can highly influence the models accuracy and performance (Fourcade et al., 2018). Maximum Entropy modelling has a variety of variable parameters that can be used to tune the model and produce a model of best fit (Phillips et al., 2009). Two of these parameters, feature class (FC) and regularisation measure (RM) are among the most useful and allow optimisation between overfitting and goodness of fit (Muscarella et al., 2014; Fourcade et al., 2018). In addition, the method of splitting training and test data for model evaluation has been shown to have strong influences on the models performance (Wenger & Olden, 2012; Bahn & McGill, 2013). One of the most common methods involves selecting a random proportion of occurrence (and background) records, usually between 20-50% as a 'pseudo-independent' test data set (Fielding & Bell, 1997). Alternative methods involve splitting the data into k number of groups (k-fold cross validation) and using each group to subsequently act as the test data, and the other groups as the training set. However, these methods of random splitting have been critised for underestimating model error and being affected by spatial autocorrelation problems (Burnham & Anderson, 2003; Araújo et al., 2005). Instead, non-random, geographical splitting of the data

may be more appropriate, and can test the extrapolation ability of the model (Radosavljevic & Anderson, 2014; Roberts et al., 2017). Initial analysis was carried out to evaluate the best method to split the test and training data, as well as the best tuning parameter combination of FCs and RMs for the baseline SDM of ancient and veteran trees across England with no bias correction method.

Section S2: Methods and analysis

MaxEnt models of ancient and veteran tree distributions across England were tuned and fitted in R (R Core Team, 2018) using the 'ENMeval' package in R (Muscarella et al., 2014). Initial model tuning using combinations of FCs 'Linear (L)', 'Linear and Quadratic (LQ)', 'Linear, Quadratic and Product (LQP)' or 'Linear, Quadratic, Product, Threshold and Hinge (LQPTH)' and RMs of 0.5, 1, 2, 3, 4, and 5 was undertaken for the model with no bias correction method applied. Model predictive power was evaluated using three methods of splitting the data into training and test data. The first method involved geographic splitting of the data into four spatial blocks, from which one was randomly assigned as test data and the others as training data ('Block'). The second method was similar but split the data into a spatial checkerboard design ('Check'), dividing the area into bins at the resolution of the raster predictors. The final method used 10 fold cross validation ('Kfold'). The splitting was carried out 10 times, with a separate model run for each, resulting in a total of 720 models (four feature classes (FC) x six regularisation methods (RM) x 3 splitting methods x 10 repetitions). Model performance was evaluated using corrected Akaike information criterion (AICc) and 'Area Under the Curve' (AUC). Generalised Linear Mixed Models (GLMMs) were used to analyse significant differences between model performance (AICc and AUC) in relation to FC, RM and splitting methods. GLMMs were fitted in R using package 'lme4' (Bates et al., 2015) separately for training and test data specifying a Gaussian distribution, and included splitting method, FC and

RM as fixed factors, and repetition run as a random factor. Backward selection based on AIC was used to find the most parsimonious model with the most influential predictors.

Section S3: Results

Model performance and predictive power differed significantly across splitting method, FC and RM (Appendix S2: Table S1). When considering each parameter separately, the most effective tuning parameters based on both mean AICc and AUC (train and test) were the 'Kfold' splitting method, FC 'LQ' and RM 5 (Appendix S2: Fig. S1 & S2). However, when considering interactions between parameters, an increase in RM only had a significantly positive influence on model performance (AICc) across FCs 'LQP' or 'LQPTH', and had little effect on model with 'L' or 'LQ' FCs (Appendix S2: Fig. S1). Therefore, the choice of RM when using either 'L' or 'LQ' FCs appears to be of little consequence, and the default version of 1 may be the best choice. Additionally, there was a significant interaction between splitting method and FC (Appendix S2: Table S1), with significantly poorer model performances with FC 'LQP' or 'LQPTH', particularly for the 'Block' splitting method (Appendix S2: Fig. S1). Therefore, based on AICc, the selection of the best tuning parameters should be based on a 'Kfold' splitting method and FC 'LQ', with any RM. When considering AUC, all parameters and interactions had a significant influence on the model predictive power (Appendix S2: Table S1). Again, the worst performing models used the 'Block' splitting method, 'LQP' and 'LQPTH' FCs and lower RMs), particularly when assessing the test data (Appendix S2: Fig. S2).

Section S4: Conclusion

The choice of tuning parameters is an important step in model fitting, as well as the division of the training and test data for model evaluation. Choice of parameters is highly model specific

and should be carried out before fitting and interpreting any SDM. In all cases, ‘Kfold’ data splitting was the most effective way to divide training and test data, regardless of any other parameter. Therefore, in all subsequent models of bias correction we have chosen to use this method. For the baseline model of ancient and veteran tree distributions with no bias correction, the combination of parameters which produce the model with both the highest performance and fit, as well as predictive power were using FC ‘LQ’ and RM 5, hence these parameters are the chosen ones for this model. For all other sampling bias corrected SDMs, models were fitted using all combinations of FC and RM as the best combination is likely to be highly variable across models. The best model for each bias correction method was then chosen based on AICc.

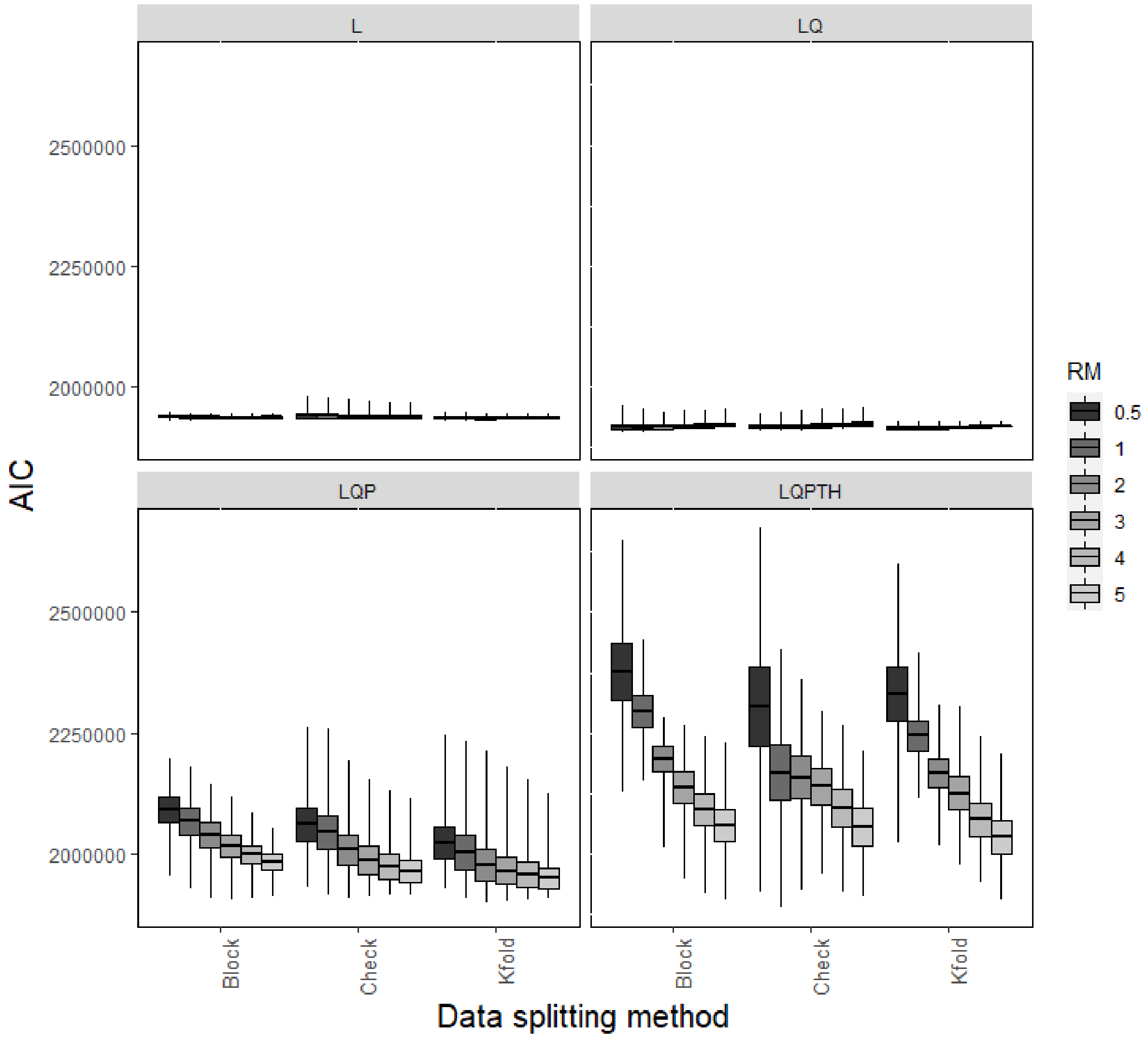

Fig. S1 *Corrected Akaike Information Criterion (AICc) for each of the model tuning combinations of splitting method into training and test data ('Block, 'Check' or 'Kfold'), feature class (FC) ('Linear (L)', 'Linear and Quadratic (LQ)', 'Linear, Quadratic and Product (LQP)' or 'Linear, Quadratic, Product, Threshold and Hinge (LQPTH)') and regularisation measure (RM) (0.5, 1, 2, 3, 4, 5). Mean values (±SE) are shown across the 10 repetitions of model fitting.*

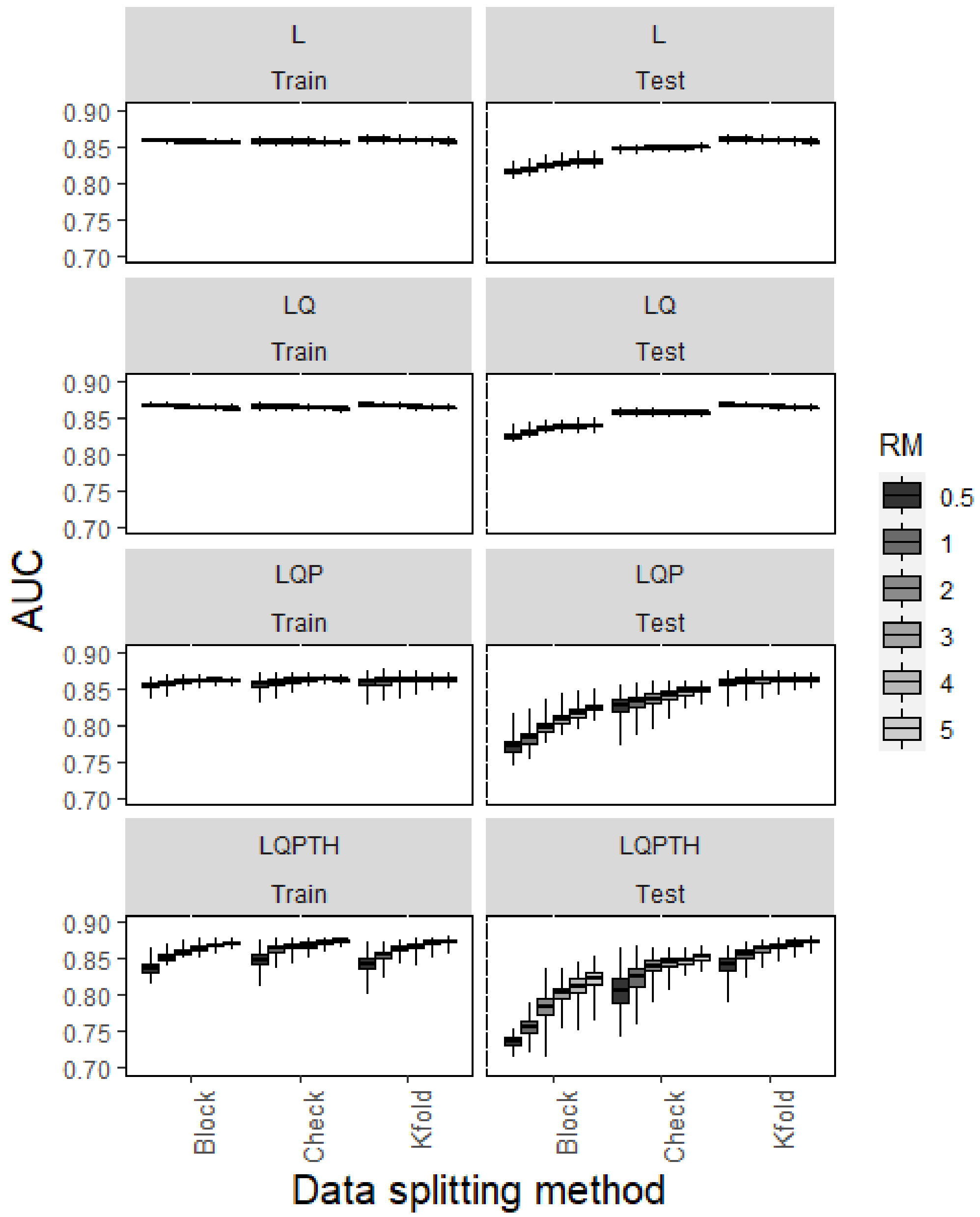

Fig. S2 *Area Under the Curve (AUC) for each of the model tuning combinations of splitting method into training and test data ('Block, 'Check' or 'Kfold'), feature class (FC) ('Linear (L)', 'Linear and Quadratic (LQ)', 'Linear, Quadratic and Product (LQP)' or 'Linear, Quadratic, Product, Threshold and Hinge (LQPTH)') and regularisation measure (RM) (0.5, 1, 2, 3, 4, 5). Mean values (±SE) are shown across the 10 repetitions of model fitting for both the training and test data set.*

Table S1 *Significance of tuning parameters in relation to model fitting (corrected Akaike Information Criterion: AICc) and model training and testing predictive power (Area Under the Curve: AUC). Parameters tested include the method of splitting data into training and testing sets ('Block', 'Check' or 'Kfold' methods), regularisation measures (RM) (0.5, 1, 2, 3, 4 and 5) and feature classes (FC) ('Linear (L)', 'Linear and Quadratic (LQ)', 'Linear, Quadratic and Product (LQP)' or 'Linear, Quadratic, Product, Threshold and Hinge (LQPTH)'). Results shown are based on a Type III analysis of variance (ANOVA) F test (with degrees of freedom) and significance levels of the p value (* < 0.05, ** < 0.01, *** < 0.001).*

	**AICc**	**AUCtrain**	**AUCtest**
RM	28.26 (5,635)***	20.18 (1,687)***	184.1 (1,687)***
FC	447.9 (3,635)***	19.77 (3,687)***	129.3 (3,687)***
Splitting method	5.915 (2,635)**	7.423 (2,687)***	358.5 (2,687)***
RM:FC	14.08 (15,635)***	21.26 (3,687)***	61.16 (3,687)***
RM: Splitting method	0.336 (10,635)	3.114 (2,687)*	48.33 (2,687)***
FC: Splitting method	2.399 (6,635)*	5.996 (6,687)***	28.97 (6,687)***
RM: FC: Splitting method	0.367 (30,635)	3.045 (6,687)**	7.450 (6,687)***

Section S5: Additional References

Araújo, M. B., Whittaker, R. J., Ladle, R. J., & Erhard, M. (2005). Reducing uncertainty in projections of extinction risk from climate change. *Global Ecology and Biogeography*, *14*(6), 529–538.

Bahn, V., & McGill, B. J. (2013). Testing the predictive performance of distribution models. *Oikos*, *122*(3), 321–331.

Burnham, K. P., & Anderson, D. R. (2003). *Model Selection and Multimodel Inference: A Practical Information-Theoretic Approach*. Springer Science & Business Media.

Fielding, A. H., & Bell, J. F. (1997). A review of methods for the assessment of prediction errors in conservation presence/absence models. *Environmental Conservation*, *24*(1), 38–49.

Fourcade, Y., Besnard, A. G., & Secondi, J. (2018). Paintings predict the distribution of species, or the challenge of selecting environmental predictors and evaluation statistics. *Global Ecology and Biogeography*, *27*(2), 245–256.

Muscarella, R., Galante, P. J., Soley-Guardia, M., Boria, R. A., Kass, J. M., Uriarte, M., & Anderson, R. P. (2014). ENMeval: An R package for conducting spatially independent evaluations and estimating optimal model complexity for Maxent ecological niche models. *Methods in Ecology and Evolution*, *5*(11), 1198–1205.

Radosavljevic, A., & Anderson, R. P. (2014). Making better Maxent models of species distributions: Complexity, overfitting and evaluation. *Journal of Biogeography*, *41*(4), 629–643.

Roberts, D. R., Bahn, V., Ciuti, S., Boyce, M. S., Elith, J., Guillera-Arroita, G., … Dormann, C. F. (2017). Cross-validation strategies for data with temporal, spatial, hierarchical, or phylogenetic structure. *Ecography*, *40*(8), 913–929.

Wenger, S. J., & Olden, J. D. (2012). Assessing transferability of ecological models: An underappreciated aspect of statistical validation. *Methods in Ecology and Evolution*, *3*(2), 260–267.
